# Patient-Reported Factors Associated With Older Adults’ Cancer Screening Decision-making: A Systematic Review

**DOI:** 10.1001/jamanetworkopen.2021.33406

**Published:** 2021-11-08

**Authors:** Jenna Smith, Rachael H. Dodd, Karen M. Gainey, Vasi Naganathan, Erin Cvejic, Jesse Jansen, Kirsten J. McCaffery

**Affiliations:** 1 Wiser Healthcare, Sydney School of Public Health, Faculty of Medicine and Health, The University of Sydney, Sydney, NSW, Australia; 2 Sydney Health Literacy Lab, Sydney School of Public Health, Faculty of Medicine and Health, The University of Sydney, Sydney, NSW, Australia; 3 Centre for Education and Research on Ageing, Concord Clinical School, The University of Sydney, Sydney, NSW, Australia; 4 School for Public Health and Primary Care, Faculty of Health, Medicine and Life Sciences, Maastricht University, Maastricht, the Netherlands

## Abstract

**Importance:**

Decisions for older adults (aged ≥65 years) and their clinicians about whether to continue to screen for cancer are not easy. Many older adults who are frail or have limited life expectancy or comorbidities continue to be screened for cancer despite guidelines suggesting they should not; furthermore, many older adults have limited knowledge of the potential harms of continuing to be screened.

**Objective:**

To summarize the patient-reported factors associated with older adults’ decisions regarding screening for breast, prostate, colorectal, and cervical cancer.

**Evidence Review:**

Studies were identified by searching databases from January 2000 to June 2020 and were independently assessed for inclusion by 2 authors. Data extraction and risk of bias assessment were independently conducted by 2 authors, and then all decisions were cross-checked and discussed where necessary. Data analysis was performed from September to December 2020.

**Findings:**

The search yielded 2475 records, of which 21 unique studies were included. Nine studies were quantitative, 8 were qualitative, and 4 used mixed method designs. Of the 21 studies, 17 were conducted in the US, and 10 of 21 assessed breast cancer screening decisions only. Factors associated with decision-making were synthesized into 5 categories: demographic, health and clinical, psychological, physician, and social and system. Commonly identified factors associated with the decision to undergo screening included personal or family history of cancer, positive screening attitudes, routine or habit, to gain knowledge, friends, and a physician’s recommendation. Factors associated with the decision to forgo screening included being older, negative screening attitudes, and desire not to know about cancer. Some factors had varying associations, including insurance coverage, living in a nursing home, prior screening experience, health problems, limited life expectancy, perceived cancer risk, risks of screening, family, and a physician’s recommendation to stop.

**Conclusions and Relevance:**

Although guidelines suggest incorporating life expectancy and health status to inform older adults’ cancer screening decisions, older adults’ ingrained beliefs about screening may run counter to these concepts. Communication strategies are needed that support older adults to make informed cancer screening decisions by addressing underlying screening beliefs in context with their perceived and actual risk of developing cancer.

## Key Points

**Question:**

What are the factors associated with older adults’ cancer screening decision-making?

**Findings:**

This systematic review of 21 studies identified that positive screening attitudes and gaining knowledge and reassurance were associated with the decision to undergo screening, whereas negative screening attitudes and not wanting to know whether one has cancer were associated with the decision to forgo screening. Other notable factors with varying associations included poor health status and health problems, limited life expectancy, and a physician’s recommendation to stop cancer screening.

**Meaning:**

Communication strategies should support older adults to make informed cancer screening decisions by addressing underlying screening beliefs in context with their perceived and actual risk of developing cancer.

## Teaser

This systematic review summarizes the patient-reported factors associated with older adults’ decisions to continue screening for breast, prostate, colorectal, and cervical cancer.

## Introduction

Decisions for older adults (aged ≥65 years) and their clinicians about cancer screening are not easy. The evidence of benefits is unclear and the chance of harm becomes greater (eg, overdiagnosis, burdens of additional testing, false-positive results, and psychological impacts),^1,2,3^ which can adversely impact an older individual’s remaining quality of life.^4,5,6,7^ Although guidelines recommend when screening is no longer needed (eg, life expectancy <10 years for breast screening),^8^ many older people with limited life expectancy continue screening for colorectal, breast, cervical, and prostate cancer.^9,10^

There has recently been a shift in cancer screening communication to support informed decision-making rather than persuasively promote uptake.^11^ Some clinicians and researchers also advocate for individualized screening decisions for older adults (ie, not solely based on age).^4,6^ However, using these approaches in practice is challenging. Clinicians have varied knowledge about overscreening and find it difficult to discuss stopping screening.^12^ Furthermore, some older adults may not appreciate a recommendation to stop screening,^13^ and positive screening attitudes may be associated with continued screening.^14^

A greater understanding of the factors associated with cancer screening decision-making will provide helpful insight for clinicians navigating screening discussions with older patients and inform the design of interventions to support informed decision-making. The aim of this review is to examine the patient-reported factors associated with older adults’ breast, prostate, colorectal, and cervical cancer screening decisions.

## Methods

### Protocol and Registration

This systematic review was registered as a protocol with PROSPERO (CRD42020185642). Reporting is guided by the Preferred Reporting Items for Systematic Reviews and Meta-analyses (PRISMA) reporting guideline.^15^

### Search Strategy

A comprehensive search strategy was developed in consultation with an academic librarian (eTable 1 in the Supplement). We searched Medline, Pre-Medline, EMBASE, PsycINFO, and CINAHL on June 2, 2020. Duplicates were removed before 2 researchers (J.S. and R.H.D.) independently screened titles and abstracts for inclusion and exclusion. Using an eligibility checklist, decisions were made independently, and disagreements were resolved via discussion. Any uncertainty that arose was taken to the research team for further discussion. Once decisions were finalized, a backward citation search and forward citation search (on January 13, 2021) was conducted.

### Inclusion and Exclusion Criteria

Studies were included if they empirically assessed patient-reported factors associated with older adults’ cancer screening decision-making (eg, patient-reported decision, choice, or intention) and included adults aged 65 years or older, a subgroup of participants aged 65 years or older or with limited life expectancy (<10 years), or participants with a mean or median age of 65 years or older (if there was also evidence of targeted recruitment of older people). Studies were included that assessed breast (mammography), prostate (prostate-specific antigen testing), colorectal (fecal occult blood testing or colonoscopy), or cervical (Papanicolaou test or human papillomavirus test) cancer screening decisions. Quantitative, qualitative, and mixed-methods peer-reviewed publications or theses were included.

Studies were excluded if they analyzed factors associated with retrospective screening or assessed decision-making regarding diagnostic or surveillance tests. To ensure that decision-making was examined in the general older population, studies that included participants at high risk or who received a diagnosis of the cancer type being examined (unless the proportion was inconsequential [<10% of the sample] or any impact on decision-making was accounted for) were excluded. Reviews, editorials, commentaries, research letters, intervention studies, or protocols were also excluded.

### Risk of Bias Assessment and Data Extraction

Risk of bias was assessed independently by 2 researchers (J.S. and K.M.G.). The Joanna Briggs Institute critical appraisal tools were used (Prevalence Studies Checklist for survey studies and Qualitative Research Checklist for interview and focus group studies).^16^ Items relating to different aspects of the methods of included studies were individually scored to determine an overall assessment of study quality (low, moderate, or high risk of bias). See eTable 2 and eTable 3 in the Supplement for more details.

### Statistical Analysis

A standardized template was developed and piloted to extract data, guided by previous systematic reviews.^17,18,19^ Data were independently extracted by 2 authors (J.S. and K.M.G.) and disagreements were discussed. Narrative synthesis was used to summarize the data. Data analysis was performed from September to December 2020.

## Results

**Figure 1.**
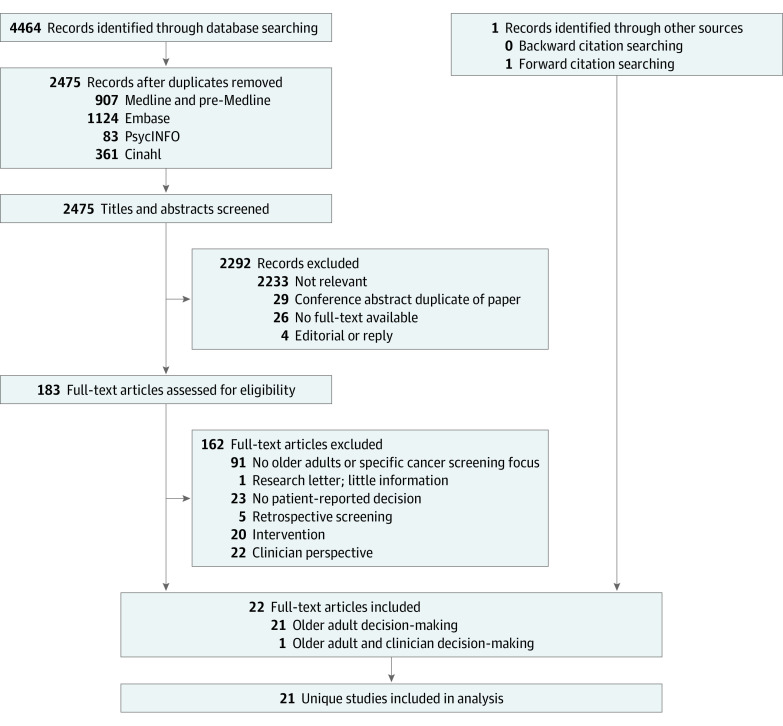
Flow Diagram of Included Studies

We screened 2475 titles and abstracts after removal of duplicates and reviewed 183 full texts, of which 22 met inclusion criteria (Figure 1). Two articles reported data from the same study,^14,20^ leaving 21 unique studies.

 Nine of the included studies were quantitative,^14,21,22,23,24,25,26,27,28^ 8 were qualitative,^13,29,30,31,32,33,34,35^ and 4 used mixed methods.^36,37,38,39^ Ten studies examined breast cancer screening,^21,22,24,25,29,30,31,32,36,39^ 3 assessed cervical cancer,^26,28,34^ 1 assessed prostate cancer,^23^ and 7 assessed some combination of breast, prostate, cervical, and colorectal cancer screening.^13,14,27,33,35,37,38^ Thirteen studies included women only,^21,22,24,25,26,28,29,30,31,32,34,36,39^ 1 included men only,^23^ and 7 included a combination of men and women.^13,14,27,33,35,37,38^ Most studies were conducted in the US (17 of 21 studies), 2 in the United Kingdom,^21,36^ 1 in Germany,^37^ and 1 in Brazil.^34^ Five studies analyzed factors associated with screening intention^21,22,24,26^ or willingness to stop,^28^ 1 assessed decisions within a discrete choice experiment for a hypothetical patient,^14^ and 1 examined factors associated with the decision process (eg, goals, concerns, and knowledge).^27^ Two studies asked closed questions about screening,^23,25^ 9 asked open questions about screening^29,31,32,34,39^ or specifically about stopping,^13,30,33,35^ and 3 asked both open and closed questions.^36,37,38^ See Table 1 for a summary of the characteristics of the included studies.

**Table 1.** Characteristics of Included Studies

Source	Country and area	Study aim	Study design and setting	Participants, No.	Age range, y (% of participants)	Description of health of sample^a^	Women, %	Cancer type	Patient-reported factors included in study	Risk of bias
Edwards et al,^21^ 2000	England and Wales	To determine breast screening uptake in older women and to ascertain from previous nonattenders whether they would accept screening if invited^b^	Cross-sectional survey; Family Health Service Authorities registers; interviewed in homes	1604	65-69 (25)	Disability: none, 21%; some, 43%; appreciable, 16%; severe, 19%	100	Breast	Demographic	Low
					70-79 (51)				Health and clinical	
					≥80 (24)					
Eisner et al,^22^ 2002	US	To identify the reasons why older women seek or do not seek mammography screening	Cross-sectional telephone survey; secondary data analysis; nationally representative households, National Cancer Institute	814	65-70 (37)	Not reported	100	Breast	Demographic	Low
					71-74 (17)				Psychological	
					75-98 (46)					
Gaehle et al,^29^ 2004^c^	Midwest US	To understand the knowledge, beliefs, and attitudes of these older women regarding breast self-examination, clinical breast examination, and mammography as breast cancer screening measures	Qualitative focus groups; convenience sampling, parish nurse organization	30	65-74 (43)	Women lived independently and indicated good functional status	100	Breast	Psychological	Low
					75-84 (43)				Physician	
					Unknown (4)				Social and system	
Gregory et al,^23^ 2007^c^	Iowa	To understand men’s decision-making process for prostate-specific antigen screening, especially among elderly men, and a more knowledgeable basis for interventions to modify screening rates	Cross-sectional postal survey; sample from Iowa’s voter registration list	452 (≥50 y)	Mean (range), 74 (50-97)	Self-reported fair-poor health: 65-74 y, 14%; 75-84 y, 26%; 85-100 y, 30%	0	Prostate	Demographic	Low
									Health and clinical	
									Psychological	
									Physician	
									Social and system	
Housten et al,^30^ 2018	Texas	To examine the willingness of older women from different racial and ethnic groups to discontinue breast cancer screening	Qualitative semistructured interviews; community outreach	29	70-74 (62)	Fair health status, 31%	100	Breast	Demographic	Low
					≥75 (38)				Health and clinical	
									Psychological	
									Physician	
									Social and system	
Madadi et al,^24^ 2014	US	To identify significant factors (sociodemographic, health related, behavioral attributes, and knowledge mammography) associated with women’s adherence to mammography screening and to study the attitudes toward mammography in nonadherent women^d^	Cross-sectional telephone survey; secondary analysis of Health Information National Trends Survey data; nationally representative	758	Subgroup ≥65 y	Not reported	100	Breast	Demographic	Low
									Health and clinical	
									Psychological	
									Physician	
Pappadis et al,^39^ 2018	Texas	To examine the perceptions of overdetection in breast cancer screening mammography and its influence on screening intentions among a triethnic sample of older women aged ≥70 y	Mixed methods; semistructured interviews; senior community living facilities, community centers, churches, local clinics	59	Mean (SD) [range], 77.5 (6.7) [70-92]	Fair-very poor health status: 30%	100	Breast	Health and clinical	Low
									Psychological	
									Physician	
					70-74 (47)					
					≥75 (53)					
Schoenborn et al,^13^ 2017	US	To examine community-dwelling older adults’ perspectives on the decision to stop cancer screening when life expectancy is limited and to identify their preferences for how clinicians should communicate recommendations to cease cancer screening.	Qualitative semistructured interviews; community-dwelling adults; clinical programs affiliated with urban academic medical center	40	Mean (range), 75.7 (65-92)	Fair-poor health status: 15%	58	Breast	Demographic	Low
						Estimated life expectancy: >10 y, 52%; 4-10 y, 28%; <4 y, 20%		Prostate	Health and clinical	
								Colorectal	Physician	
Schoenborn et al,^14^ 2019	US	To identify the factors that are best at estimating decisions for breast cancer, colorectal cancer, and prostate cancer screenings in older adults; data same as in Janssen et al^20^; different analysis	Discrete choice experiment; KnowledgePanel members surveyed online, invited via email	881	Mean (SD), 73.4 (6.1)	Estimated life expectancy <10 y, 31%	55.2	Breast	Demographic	Low
								Prostate	Health and clinical	
								Colorectal	Psychological	
									Physician	
Schonberg et al,^31^ 2006	US	To explore decision-making and physician counseling of oldest-old women around mammography screening	Qualitative semistructured interviews; academic primary care practice	23	Mean (SD) [range], 86 (4) [80-97]	IADL dependence: 7	100	Breast	Demographic	Low
						ADL and IADL dependence: 7			Health and clinical	
						Fair-poor health status: 10			Psychological	
									Physician	
									Social and system	
Schonberg et al,^25^ 2007	Boston, MA	To identify factors important to mammography screening decisions among women aged 65-79 y vs those aged ≥80 y	Cross-sectional telephone survey; academic primary care practice	200	65-79 y: mean, 71.5 y	65-79 y: CCI score 0, 49%; CCI score 1, 29%; CCI score ≥2, 22%; IADL dependence, 13%; fair-poor health status, 13%^e^	100	Breast	Demographic	Low
					≥80 y: mean, 85.3 y	≥80 y: CCI score 0, 46%; CCI score 1, 22%; CCI score ≥2, 34%; IADL dependence, 37%; fair-poor health status,^e^ 21%			Health and clinical	
									Psychological	
									Physician	
									Social and system	
Swinney et al,^32^ 2011	US	To identify social, cultural, and behavioral factors associated with regular participation in breast cancer screening; examine health beliefs that may influence regular participation; and identify perceived facilitators and barriers	Qualitative focus groups; community	57	Mean (SD) [range], 80 (9.09) [65-94]	100% Self-reported as healthy despite receiving treatment	100	Breast	Psychological	Low
						Diabetes: 6			Social and system	
						Cardiac conditions: 4				
						Hypertension: 12				
Torke et al,^33^ 2013	US	To obtain a deeper understanding of older adults’ perspectives on screening cessation and their experiences of communication with clinicians about this topic	Qualitative semistructured interviews; senior health center	33	Median (range), 76 (63-91)	Mild cognitive impairment: 42%	82	Breast	Demographic	Low
						Chronic medical conditions: mean 2.5		Prostate	Health and clinical	
					Targeted recruitment			Colorectal	Psychological	
						Personal history of cancer: 2^e^			Physician	
									Social and system	
Zhang et al,^26^ 2007	US	To examine the relative importance of objective (eg, intact cervix) and subjective factors (eg, patients’ Papanicolaou test related beliefs and attitudes) as correlates of elderly women’s intention to have a Papanicolaou test	Cross-sectional telephone survey; secondary data analysis; Texas Tech 5000 Survey wave 4	1037	Mean (SD), 74.85 (6.08)	NA	100	Cervical	Demographic	Low
									Health and clinical	
					65-70 (32)				Psychological	
					71-75 (31)					
					76-80 (20)					
					≥81 (17)					
Collins et al,^36^ 2010	United Kingdom	To examine the views, knowledge and attitudes of older women (>70 y) toward mammographic breast screening	Mixed methods; semistructured interviews, outpatient clinics in hospital; postal questionnaire, sampled from general practitioner practice	26 Interviews; 469 survey	Median (range), 75 (70-95)	88% Reported ≥1 long-term health problem^e^	100	Breast	Demographic	Low to moderate
									Health and clinical	
					70-74 (43)	76% Functionally independent			Psychological	
					75-79 (29)				Social ad system	
					80-84 (15)					
					≥85 (13)					
Lewis et al,^38^ 2006	North Carolina	To determine if older adults reported the opportunity to engage in individualized decision-making with physicians, attitudes about information important for individualized decision-making and continuing screening later in life	Mixed methods; face to face survey with closed and open-ended questions; retirement communities	116	Mean (SD), 81.5 (5.2)	Independent in ADLs: 95%	67	Breast	Demographic	Low to moderate
					70-79 (36)	Fair health status: 9%^e^		Prostate	Health and clinical	
					80-84 (33)				Psychological	
					≥85 (31)	CCI score 0, 30%; score 1, 30%; score 2-4, 40%		Colorectal	Physician	
									Social and system	
Fairfield et al,^27^ 2015	US	To describe decision process and quality for common cancer screening decisions by age group; self-reported quality of decision-making processes, importance of specific goals and concerns, and knowledge	Cross-sectional internet survey; probability-based Knowledge Networks internet sample	2941 (≥40 y)	Subgroup ≥75 y	Not reported for ≥75 subgroup	Not reported	Breast	Physician	Moderate
								Prostate		
								Colorectal		
Oliveira Leite et al,^34^ 2019	Montes Claros, Brazil	To describe the perceptions of older women about cancer-preventive cervical examination	Qualitative semistructured interviews; Vila Analia Family Health Strategy; in home	12	Range, 65-93	Not reported	100	Cervical	Psychological	Moderate
									Physician	
Roy et al,^35^ 2020	Pennsylvania	To characterize how older adults from underserved backgrounds perceive cancer screening and overscreening	Qualitative focus groups; English- and Spanish-speaking older adults; community and senior centers, retirement communities	39	Mean 74	Not reported	74	Breast	Health and clinical	Moderate
					≥65			Cervical	Psychological	
								Colorectal	Physician	
									Social and system	
Sawaya et al,^28^ 2009	US	To examine attitudes and beliefs on ending cervical cancer screening from an ethnically diverse group of women aged ≥65 y	Cross-sectional face to face survey; primary care practices	199	Mean, 70.9	Fair-poor health status: 46%	100	Cervical	Demographic	Moderate
					65-69 (44)				Health and clinical	
					70-74 (36)				Psychological	
					≥75 (20)				Physician	
Dolezil et al,^37^ 2016	Northeast Germany	To investigate the attitudes of older adults toward cancer screening as well as their motives for or against participation	Mixed methods; face-to-face surveys and semistructured interviews; sampled via mail from population register	120 Surveys; 46 interviews	Mean (SD), 77 (6)	CCI score 0, 67%; CCI score 1, 23%; CCI score 2-5, 10%	47	General (statements re: breast, prostate, colorectal)	Demographic	Moderate to high
					69-74 (46)				Health and clinical	
					75-79 (24)				Psychological	
					80-84 (15)				Physician	
					85-90 (15)					

Abbreviations: ADL, activities of daily living; CCI, Charlson Comorbidity Index; IADL, instrumental activities of daily living.

^a^ Self-reported health status response options include excellent, very good, good, fair, poor. For the CCI, a higher score indicates more comorbidities.

^b^ Edwards et al^21^ only examined factors associated with future uptake of screening in those who previously had not attended.

^c^ Article is a thesis.

^d^ Madadi et al^24^ analyzed data from the Health Information National Trends Survey, which measured intention to screen and defined mammography adherence as positive screening intentions and attitudes.

^e^ Study included some participants with cancer diagnosis. Collins et al^36^ and Lewis et al^38^ found no differences in responses between those with and without cancer history. Schonberg et al^25^ incorporated personal cancer history as potential factor associated with decision-making in survey design. Torke et al^33^ included only 2 of 33 participants with personal history of cancer.

Most studies included adults aged 65 years or older (17 of 21 studies),^13,14,21,22,25,26,28,29,30,31,32,34,35,36,37,38,39^ 3 included a subgroup of adults aged 65 years or older,^23,24,27^ and 1 study reported a mean or median age 65 years or older and targeted recruitment via a senior health center.^33^ Most had representation from younger (65-74 years) and older (≥75 years) age ranges,^13,14,21,22,23,25,26,28,29,30,32,33,34,35,36,37,38,39^ except for 3 studies: 2 included only adults aged 75 years or older^27^ or 80 years or older,^31^ and 1 did not report an age range.^24^ Many studies also included participants with self-reported fair to poor health,^13,23,28,30,31,39^ 2 or more health problems or comorbidities,^25,32,36,38^ mild cognitive impairment,^33^ life expectancy less than 10 years,^14^ or appreciable or severe physical disability.^21^ Two studies included mostly healthy participants,^29,37^ and 6 did not report health characteristics.^22,24,26,27,34,35^ Four studies included participants with a personal history of cancer (see Table 1 for reasons for inclusion).^25,33,36,38^

**Figure 2.**
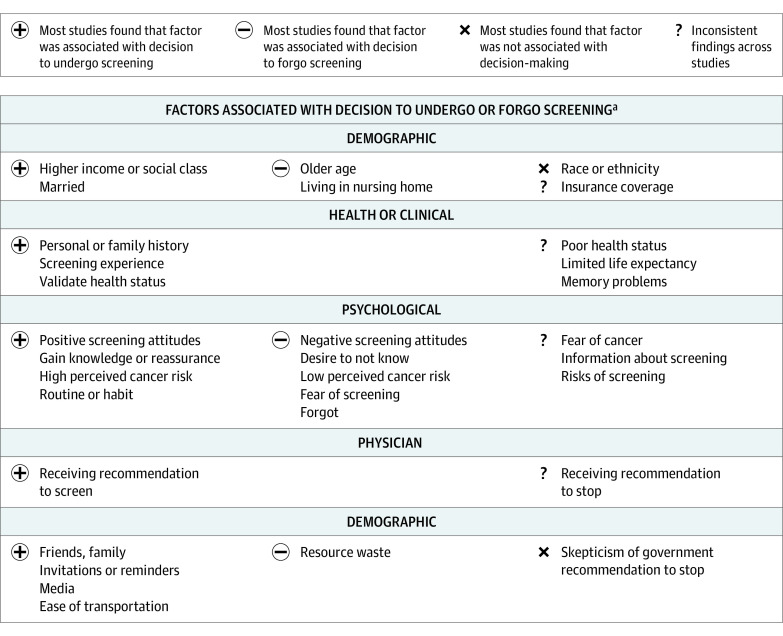
Summary of Factors Associated With Older Adults’ Cancer Screening Decision-making ^a^Factors are included only if they are reported in more than 1 study.

Risk of bias assessment is reported in the eAppendix in the Supplement and is incorporated throughout the results for studies that report conflicting or stand-alone findings. Main findings from the included studies are categorized and synthesized narratively below with additional details presented in Table 2 (quantitative results) and Table 3 (qualitative results). Figure 2 shows a summary of factors associated with older adults’ cancer screening decision-making.

**Table 2.** Summary of Quantitative Studies Examining Factors Associated With Cancer Screening Decision-making

Factor and source	Outcome and analysis details	Variable	Results and statistics
Demographic			
Collins et al,^36^ 2010	Closed questions about screening in women aged ≥70 y	Age	90.1% Agreed that breast screening should be offered to all women indefinitely and regardless of age; 42.9% indicated preference for automatic recall extended indefinitely regardless of age; 18.8% of nonattenders felt mammograms not needed after age 70 y
Dolezil et al,^37^ 2016	Closed questions about utilization of early cancer screening	Living in nursing home	12% Agreed or strongly agreed that cancer screening should no longer be performed for people >70 y old in nursing homes
Edwards et al,^21^ 2000	Of those who had not attended, percentage who would uptake breast screening in future; χ^2^ tests; unadjusted values only	Age	65-69 y, 67%; 70-79 y, 53%; ≥80 y, 27%; *P* < .001
		Social class	Highest, 62%; lowest, 35%; *P* < .05
		Marital status	Married, 60%; single, 40%; separated or divorced, 52%; widowed, 43%; *P* < .001
		Living arrangements	Alone, 46%; spouse only, 61%; spouse and others, 54%; others, 36%; *P* < .001
Eisner et al,^22^ 2002	Percentage planning to have mammogram in next year; Neuman-Kuels test	Age	65-69 y, 80%; ≥70 y, 70%; *P* < .05
	Unadjusted values only	Insurance (paid for last mammogram with Medicare)	Yes, 85%; no, 74%; *P* > .05
Gregory et al,^23^2007	Factors likely to influence undergoing PSA screening	Insurance	49% Indicated likely to influence undergoing PSA screening; no difference across age subgroups (50-64, 65-74, 75-84, or 85-100 y)
Lewis et al,^38^ 2006	Closed questions about continuing screening later in life	Living in nursing homes	When considering others, most believed that those living in nursing homes (74%) should continue to get screened
Madadi et al,^24^ 2014	Mammography screening adherence (positive attitudes and intentions); adjusted values only	Race	Black (reference), White (OR, 1.329; *P* = .536), other (OR, 0.425; *P* = .148)
		Marital status	Married (reference), single, divorced, or widowed (OR, 0.499; *P* = .012)
		Education	Less than high school (reference), high school (OR, 1.460; *P* = .310), some college or college graduate (OR, 1.507; *P* = .320)
		Annual income	<$25 000 (reference), $25 000-75 000 (OR, 1.341; *P* = .044), >75 000 (OR, 1.992; *P* = .609)
		Insurance	No (reference), yes (OR, 2.771; *P* = .130)
Sawaya et al,^28^ 2009	Percentage who would stop Papanicolaou tests after recommendation from physician; χ^2^ test; multivariable regression; unadjusted values and adjusted where significant	Age (continuous)	65-69 y, 55.1%; 70-74 y, 74.7%; ≥75 y, 88.5% (adjusted OR, 1.25; 95% CI, 1.09-1.44; *P* = .002)
		Marital status	Married, 62.8%; formerly married, 73.5%; never married, 64.3%; *P* = .333
		Education	Less than high school, 81.7%; high school or some college, 57.5%; college or graduate school, 59.7%; *P* = .006
		Insurance	Public, 79.4% (adjusted OR, 3.84; 95% CI, 1.56-9.46); private, 52.9% (reference); *P* < .001 (unadjusted)
		Annual income	≤$15 000, 78.6%; >$15 000, 61.6%; *P* = .051
		Ethnicity (country of birth)	Born in US, 56.5%; born outside US, 74.1%; *P* = .017 (adjusted *P* = .051)
Schoenborn et al,^14^ 2019	Discrete choice experiment; hypothetical screening test	Age of hypothetical patient	Of those with positive screening attitudes, age was third most associated factor (first, screening attitudes; second, life expectancy); of those without positive screening attitudes; age was second most associated factor; more likely to choose screening when the hypothetical patient was younger
Schonberg et al,^25^ 2007	Importance of factors in decision; subgroups 65-79 vs ≥80 y	Age	65-79 y: screening (n = 82), 28.2% vs no screening (n = 11), 50% (3/6); ≥80 y: screening (n = 80), 29.0% vs no screening (n = 26), 56.5% (13/23)
Zhang et al,^26^ 2007	Intent to have a Papanicolaou test; unadjusted logistic regression	Age	65-70 y (reference); 71-75 y (OR, 1.08; 95% CI, 0.65-1.79); 76-80 y (OR, 0.37; 95% CI, 0.21-0.65; *P* < .01); ≥81 y (OR, 0.41; 95% CI, 0.22-0.68; *P* < .01)
		Insurance coverage	Not covered Papanicolaou test (reference), insurance covered test (OR, 1.79; 95% CI, 1.16-2.77; *P* < .01)
Health and clinical			
Collins et al,^36^ 2010	Closed questions about screening in women aged ≥70 y	Health status	90.1% Agreed breast screening should be offered to all women indefinitely and regardless of health status or fitness; no association between functional status or long-term health problems and desire to continue screening after age 70 y if invited
			17.2% Of nonattenders felt other health problems seem more important.
Dolezil et al,^37^ 2016	Closed questions about utilization of early cancer screening	Life expectancy	70% Believed the assessment of their life expectancy was not important for decision about participation in screening; 35% responded true or partly true to “I will not live long enough to benefit from a cancer screening test”
		Health problems	25% Agreed or strongly agreed that as people get older, other health problems are more important than cancer screening
		Need care	35% Agreed or strongly agreed that cancer screening should no longer be performed for people >70 y old in need of care
		Dementia	21% Agreed or strongly agreed that cancer screening should no longer be performed in people who have Alzheimer disease or any other dementia
Edwards et al,^21^ 2000	Percentage who would uptake breast screening in future; χ^2^ tests; unadjusted values only	Anxiety	Yes, 56%; no, 49%; *P* < .05
		Depression	Yes, 43%; no, 51%; *P* < .05
		Disability	None, 58%; some, 54%; appreciable, 47%; severe, 34%; *P* < .0001
Eisner et al,^22^ 2002	Percentage planning to have mammogram in next year; Neuman-Kuels test	Risk factors	57% Indicated that women should continue having mammograms even when there are no risk factors
			33% Felt that women without risk factors can be less concerned about getting a mammogram
		Screening history	81% Of respondents who received their most recent mammogram in the past 2 y stated their intention to get another one within the coming year
			Women who had not had mammogram in past 2 y were more likely than those who had one to say they would never have another mammogram (32% vs 2%); they would have one 3-4 y from now (3% vs 0.2%); or they were unsure when they would have their next mammogram (13% vs 3%)
Gregory et al,^23^ 2007	Factors likely to influence receiving PSA screening	Other health problems	42% Indicated other health problems likely to influence receiving PSA screening; did not differ significantly across 4 age groups
Lewis et al,^38^ 2006	Closed questions about continuing screening later in life	Life expectancy	62% Did not think physician’s life expectancy estimate was important in making cancer screening decisions
		Other health problems	81% Agree with “I will likely die of some other disease besides cancer”
			50% Agree with “As people get older, other health issues are more important than cancer screening”
			44% Agree “People over 70 who are totally dependent on someone else for daily functions such as eating, bathing, and toileting should not get cancer screening”
			44% Agree “People with Alzheimer's disease or dementia should not get cancer screening”
Madadi et al,^24^ 2014	Mammography screening adherence (positive attitudes and intentions)	Screening history (Papanicolaou test)	No (reference) vs yes (OR, 3.809; *P* = .001)
		Exercise	Yes (reference) vs no (OR, 1.134; *P* = .623)
	Multiple logistic regression results	Screening history (mammogram frequency)	Every 1-2 y (reference); other (OR, 0.636, *P* = .048)
		No. of visits to health care practitioner/y	Adjusted: <2/y, 2-4/y (OR, 1.016; *P* = .969), ≥5/y (OR, 1.610; *P* = .313)
Sawaya et al,^28^ 2009	Percentage who would stop Papanicolaou tests after recommendation from physician; χ^2^ test; multivariable regression	Health status	Poor or fair, 72.6%; good or very good or excellent, 63.3% (adjusted); *P* = 1.902 (unadjusted)
		Family history of cancer	Yes, 55.3% (reference); no, 73.9%; OR, 3.06 (95% CI, 1.19-7.89) (adjusted); *P* = .015 (unadjusted)
		Personal history of cancer	Yes, 47.1% (reference); no, 73.1%; OR, 3.13 (95% CI, 1.12-8.73) (adjusted); *P* = .004 (unadjusted)
Schoenborn et al,^14^ 2019	Whether they would choose to get hypothetical screening test (colorectal, prostate, or breast)	Life expectancy hypothetical patient	Positive screening attitudes: second most associated with decisions; patient 1-y life expectancy, 57.2% chose screening; 1-y life expectancy and age ≥75 y, 45.8%
		Screening history (ever)	Not positive screening attitudes: third most associated; patients with prior screening were more likely to screen
		Quality of life hypothetical patient	Not positive screening attitudes: fourth most associated; good or medium, more likely to choose screening vs poor quality of life
Schonberg et al,^25^ 2007	Importance of factors in decision; subgroups 65-79 vs ≥80	History of breast disease	65-79 y: screening (n = 20), 65% vs no screening, NA; ≥80 y: screening (n = 28), 85.7% vs no screening, NA
		Family history	65-79 y: screening (n = 82), 42.3% vs no screening, NA; ≥80 y: screening (n = 80), 38.1% vs no screening, NA
		Health	65-79 y: screening (n = 82), 36.6% vs no screening, 50% (3/6); ≥80 y: screening (n = 80), 47.1% vs no screening, 19.1% (4/21)
		Previous experience with mammography	65-79 y: screening, NA vs no screening, 28.6% (2/7); ≥80 y: screening, NA vs no screening, 18.8% (3/16)
Zhang et al,^26^ 2007	Intent to have a Papanicolaou test; unadjusted logistic regression	Screening history (Papanicolaou test)	Have not had one within 2 y (reference) vs have had one (OR, 20.02; 95% CI, 13.05-30.72); *P* < .01
		Hysterectomy	Yes (reference) vs no (OR, 2.21; 95% CI, 1.47-3.31); *P* < .01
Psychological			
Collins et al,^36^ 2010	Closed questions about screening in women ≥70 y	Perceived risks	5.5% Aware of possible risks of screening, 99.2% were not worried by possible health risks associated with having a mammogram
		Lack of knowledge	35.1% Of those who did not screen said they did not know they could refer themselves
		Preferences	12.3% Of those who did not screen said they did not want any more mammograms, 4.5% thought mammograms painful or unpleasant, 3.9% worried about getting to screening center
		Forgot	9.2% Of those who did not screen said they forgot
		Embarrassment and privacy	Privacy (11.8%) and embarrassment (7.8%) were relatively uncommon reasons for not wishing to be screened
Dolezil et al,^37^ 2016	Closed questions about utilization of early cancer screening	False positives	80% Of responders also wanted to be informed about the possibility of a false-positive finding in early cancer screening
		Attitudes	75% Agreed with “As long as I live, I will take part in bowel cancer screening”
			82% Agreed with “As long as I live, I will take part in breast cancer screening”
			8% Agreed with “As long as I live, I will take part in prostate cancer screening”^a^
			8% Agreed with “In my opinion, cancer screening tests are of no use”
			73% Agreed with “Everyone should have colon cancer screening for the rest of their lives”
			89% Agreed with “Every woman should be screened for breast cancer for the rest of her life”
			86% Agreed with “Every man should be screened for prostate cancer for the rest of his life”
Eisner et al,^22^ 2002	Percentage planning to have mammogram in next year; Neuman-Kuels test	As concerned about getting breast cancer as when younger	Yes, 80% vs no, 64%; *P* < .05
		Aware of Medicare coverage	Yes, 75% vs no, 64%; *P* < .05
Fairfield et al,^27^ 2015	Rated importance of decision-specific goal and concerns (scale 0-10)	Peace of mind	Breast cancer screening: mean 9.8, *P* = .005
			Prostate cancer screening: mean 9.5, *P* = .001
			*P* value relates to difference between elderly group and younger group (60-74 y)
Gregory et al,^23^ 2007	Factors likely to influence receiving PSA screening; analysis by age subgroups: 50-64, 65-74, 75-84; mean scores are on a scale of −3 to 3 where higher values denote greater agreement	Importance of early detection	94% Agreed screening would help detect cancer early and 92% agreed this was important to them; extent of agreement was higher among men aged 65-74 y vs men age 50-64 y (mean [SD], 2.3 [1.2]); extent of agreement was higher among men aged 75-84 y (mean [SD], 2.6 [1.2]) vs men aged 50-64 y
		Peace of mind	84% Agreed screening would provide peace of mind and 86% agreed this was important to them; extent of agreement was higher among men aged 65-74 y vs men aged 50-64 y; extent of agreement was higher among men aged 75-84 y (mean [SD], 2.3 [1.5]) vs men aged 50-64 y
		Provides knowledge	90% Agreed screening would provide them knowledge of their PSA value status and 85% agreed this was important to them; extent of agreement was higher among men aged 65-74 y vs men aged 50-64 y; extent of agreement was higher among men aged 75-84 y (mean [SD], 2.3 [1.5]) vs men aged 50-64 y
		False positives	60% Agreed screening could provide false result and 79% agreed this was important to them; did not differ across the 4 age groups.
		Information or education	70% Indicated information or education about PSA screening was likely to influence receiving PSA screening; the perceived extent information about PSA screening was likely to influence screening decisions did not differ significantly across the 4 age groups
		Routine physical examination	79% Indicated including it in a routine physical examination was likely to influence receiving PSA screening; did not differ significantly across the 4 age groups
Lewis et al,^38^ 2006	Closed questions about continuing screening later in life	Attitudes	72% Agree with “I plan to get screened for colon cancer for as long as I live”
			83% Agree with “I plan to get screened for breast/prostate cancer for as long as I live”
			77% Agree with “I will continue cancer screening no matter how uncomfortable the tests are”
			25% Agree with “It takes several years for cancer screening to benefit people”
			13% Agree with “I will not live long enough to benefit from cancer screening tests”
			3% Agree with “Cancer screening is not worth the trouble”
			55% Agree with “Everyone should get screened for colon cancer for as long as they live”
			63% Agree with “Everyone should get screened for breast/prostate cancer for as long as they live”
Madadi et al,^24^ 2014	Mammography screening adherence (positive attitudes and intentions)	Looking for cancer information	Adjusted: no (reference) vs yes (OR, 0.883; *P* = .484)
	Multiple logistic regression	Perceived risk of breast cancer	Adjusted: low (reference), moderate (OR, 1.645; *P* = .054), high (OR, 3.036; *P* = .043)
		Age women should start getting mammography	Adjusted: 40-50 y (reference) vs other answers (OR, 0.692; *P* = .101)
Pappadis et al,^39^ 2018	Breast cancer screening intentions; χ^2^ test	Overdetection understanding	Women who did not understand were more likely to desire to continue screening (62% vs 37%; *P* = .045)
Sawaya et al,^28^ 2009	Percentage who would stop getting Papanicolaou tests based on physician recommendation; χ^2^ test and multivariable regression	Perceived risk of cervical cancer	No risk, very or somewhat low, 100 (69.0%); moderate or very high, 16 (59.3%); *P* = .323
Schoenborn et al,^14^ 2019	Whether they would choose to get hypothetical screening test	Screening attitudes	Most associated factor of whether a participant chose breast, colorectal, or prostate cancer screening in the vignettes
Schonberg et al,^25^ 2007	Importance of factors in decision; subgroups 65-79 y vs ≥80 y	Habit	65-79 y: screening (n = 82), 76.2% vs no screening, 50% (3/6); ≥80 y: screening (n = 80), 87.0% vs no screening, 40% (8/20)
		Reassurance	65-79 y: screening (n = 82), 81.0% vs no screening, NA; ≥80 y: screening (n = 80), 73.0% vs no screening, NA
		Not concerned about breast cancer	65-79 y: screening, NA vs no screening, 83.3% (5/6); ≥80 y: screening, NA vs no screening, 80.0% (16/20)
Zhang et al,^26^ 2007	Intent to have a Papanicolaou test; unadjusted logistic regression; reference for each is disagree	Perceived negative impact	Have trouble getting insurance coverage (OR, 0.81; 95% CI, 0.52-1.28)
		Emotional barriers	Do not like to have test (OR, 0.60; 95% CI, 0.38-0.95; *P* < .05); painful (OR, 0.59; 95% CI, 0.37-0.93; *P* < .05); bothered by the thought (OR, 1.41; 95% CI, 0.84-2.36); unpleasant or embarrassing (OR, 1.25; 95% CI, 0.77-2.04); do not like to visit gynecologist (OR, 0.78; 95% CI, 0.48-1.26); health care is unpleasant (OR, 0.92; 95% CI, 0.58-1.44)
		Cancer anxiety	Afraid that something wrong will be detected (OR, 1.16; 95% CI, 0.69-1.95); uneasy talking about cancer (OR, 1.07; 95% CI, 0.58-1.96)
		Perceived importance	Papanicolaou test is important (OR, 2.55; 95% CI, 1.15-5.69; *P* < .05); cancer screening is useful (OR, 1.09; 95% CI, 0.55-2.17)
		Perceived risk	Likely to develop cervical cancer (OR, 3.19; 95% CI, 1.26-8.08; *P* < .05); more likely to develop cervical cancer than others (OR, 0.65; 95% CI, 0.18-2.40)
Physicians			
Dolezil et al,^37^ 2016	Closed questions about utilization of early cancer screening	Discussion with physician	82% Had wanted to speak about their participation before screening and were convinced their physician could assess whether people older than 69 could benefit from screening
		Physician recommendation	6% Agree with “I won’t have a cancer screening test even if my doctor recommends it”
Fairfield et al,^27^ 2015	Percentage who decided no colorectal cancer screening	Discussion about cons	Some or a lot, 45% vs none or a little, 7%; *P* = .001 (unadjusted)
Gregory et al,^23^ 2007	Factors likely to influence receiving PSA screening; mean scores are on a scale of 1-7, where higher scores suggest greater reported influence on decision	Physician influence	71% Indicated their regular physician influences their decision very much; perceived influence higher among men aged 65-74 y (mean [SD], 6.5 [1.1]) vs men aged 50-64 y; perceived influence higher among men aged 75-84 y vs men aged 50-64 y; perceived influence greater among men aged 75-84 y vs men aged 85-100 y (mean [SD], 6.3 [1.5])
		Urologist influence	76% Indicated their urologist influences their decision very much; perceived influence higher among men aged 65-74 y vs 50-64 y
Lewis et al,^38^ 2006	Closed questions about continuing screening later in life	Physician recommendation	43% Agree with “I will consider getting screened for cancer even if my doctor recommends against it”
			4% Agree with “I will not get cancer screening even if my doctor recommends it”
		Discussion with physician	94% Agree with “I want my doctor to talk with me about how tests for cancer can give the wrong result”
			84% Agree with “I want my doctor to talk with me about whether I want to stop getting tests to check for cancer”
			52% Agree with “To help make cancer screening decisions, I want my doctor to talk with me about how long he/she thinks I might live”
			49% Agree with “I think that doctors know for sure if cancer screening helps people over 70”
Madadi et al,^24^ 2014	Mammography screening adherence (positive attitudes and intentions); multiple logistic regression	Being advised to have a mammography	Adjusted: no (reference) vs yes (OR, 10.711; *P* < .001)
Sawaya et al,^28^ 2009	Percentage who would stop getting Papanicolaou tests based on physician recommendation; χ^2^ test and multivariable regression	Important decisions should be made by physicians, not patients	Strongly or somewhat disagree, 37 (59.7%); somewhat or strongly agree, 82 (72.6%); *P* = .08
		Trust physicians make best decisions on patients’ behalf	Not at all, a little, or somewhat, 20 (71.4%) vs mostly or completely, 99 (67.8%); *P* = .705
Schoenborn et al,^14^ 2019	Whether they would choose to get hypothetical screening test	Physician recommendation	Compared with screening attitudes, age, life expectancy and quality of life, physician recommendation least associated with screening decision
Schonberg et al,^25^ 2007	Importance of factors in decision; subgroups 65-79 y vs ≥80 y	Physician recommendation	65-79 y: screening (n = 82), 60.2% vs no screening, 28.6% (2/7); ≥80 y: screening (n = 80), 66.2% vs no screening, 50.0% (11/22)
Zhang et al,^26^ 2007	Intent to have a Papanicolaou test; unadjusted logistic regression; reference for each is disagree	Physician recommendation	Physician feel having test is good idea now (OR, 5.58; 95% CI, 3.20-9.70; *P* < .01)
Social and system			
Collins et al,^36^ 2010	Closed questions about screening in women ≥70 y	Invitation	61.6% Would forget to attend screening without an invitation; 74.1% prefer a reminder letter every 3 y to prompt them to attend; 52.1% of nonattenders’ reason was that they were not invited for screening so thought not necessary
		Transport	25.6% Discouraged from attending because of transport difficulties (either public transport, parking problems)
		Burden to family	24.7% Discouraged from attending because they do not wish to burden family members
Dolezil et al,^37^ 2016	Closed questions about utilization of early cancer screening	Wastes resources	5% Agreed or strongly agreed that cancer screening for people older than 70 y is a waste of time and money
Gregory et al,^23^2007	Factors likely to influence receiving PSA screening; analysis by age subgroups: 50-64, 65-74, and 75-84 y; mean scores are on a scale of 1-7	Wife influence	58% Indicated their wife influences their decision to screen in the next year very much and 6% not at all; perceived influence greater among men aged 65-74 y vs 50-64 y; perceived influence greater among men aged 75-84 y (mean [SD], 6.1 [1.8]) vs 50-64
		Family or friends influence	40% Indicated family or friends who had cancer influence their decision in the next year very much; perceived influence higher among men aged 65-74 y vs 50-64 y; 44% indicated their family influences their decision to get screening in the next year very much; perceived influence higher among men aged 65-74 y vs 50-64 y; the perceived influence higher among men aged 75-84 y (mean [SD], 5.8 [1.9]) vs 50-64 y; the perceived influence higher among men aged 85-100 y (mean [SD], 5.6 [1.8]) vs 50-64 y
			26% Indicated their friends influence their decision very much; perceived influence did not differ across the 4 age groups
		Transportation	26% Indicated transportation or the distance required to receive PSA screening was likely to influence receiving PSA screening; perceived extent transportation and/or distance would influence screening decisions did not differ significantly across the 4 age groups
Lewis et al,^38^ 2006	Attitudes toward continuing screening later in life	Wastes resources	30% Agree with “Screening for cancer in people over the age of 70 may waste healthcare time and money”
Schonberg et al,^25^ 2007	Asked how important factors were in their decision to screen (or not) in past 2 y; subgroups 65-79 y vs ≥80 y	Reminder card	65-79 y: screening (n = 62), 72.6% vs no screening, NA; ≥80 y: screening (n = 38), 73.7% vs no screening, NA
		Friend’s experience with breast cancer	65-79 y: screening (n = 82), 30.8% vs no screening, NA; ≥80 y: screening (n = 80), 25.0% vs no screening, NA
		Family recommendation	65-79 y: screening (n = 82), 15.9% vs no screening, NA; ≥80 y: screening (n = 80), 13.9% vs no screening, NA
		Friend recommendation	65-79 y: screening (n = 82), 16.9% vs no screening, NA; ≥80 y: screening (n = 80), 6.3% vs no screening, NA
		Media	65-79 y: screening (n = 82), 15.5% vs no screening, 0% (0/6); ≥80 y: screening (n = 80), 16.0% vs no screening, 8.3% (2/24)
Zhang et al,^26^ 2007	Intent to have a Papanicolaou test; unadjusted logistic regression; reference for each is disagree	Perceived positive impact for family	Give family useful information (OR, 1.59; 95% CI, 0.724-3.48); help family make decision (OR, 1.78; 95% CI, 0.78-4.03)

Abbreviations: NA, not applicable; OR, odds ratio; PSA, prostate-specific antigen.

^a^ As presented in original article; however, it was assumed to be a typographical error considering the high percentages for other cancer types. We were unable to contact the author for clarification.

**Table 3.** Summary of Qualitative Studies Examining Factors Associated With Cancer Screening Decision-making

Factor and source	Outcome and analysis details	Findings and quotations
Demographic		
Collins et al,^36^ 2010	Open questions about screening in women ≥70 y	Age: “Why 73?...Well you’re on the scrap heap…I think it should be for everybody whatever age, however old, whatever their health.”
Housten et al,^30^ 2018	Circumstances that would lead them to stop having mammograms	Living in a nursing home: more than half of participants stated living in a nursing home would not prevent them from continuing to get mammograms. “Yes, if they thought it [mammogram] was a routine, a precaution or early detection of anything. I wouldn’t care if I was 100. If they thought well this [mammogram] is something we still do because it’s [cancer] still happening to older ladies, I would say go ahead, do it.”
Lewis et al,^38^ 2006	Attitudes toward continuing screening later in life	Age: “I am ninety-two and I don't intend to prolong this if I don’t have to.” “If I got to be really old, I think I would say to heck with it. Like in my nineties.”
Schoenborn et al,^13^ 2017	Explored considerations around the decision to stop screening	Age: In addition to the participants who already decided to stop screening, others mentioned hypothetical scenarios in which they would consider screening cessation. Older age was the most common reason; one 84-y-old woman said, “I just feel like at my age I don’t need a colonoscopy, what’s gonna be is gonna be.”
Schonberg et al,^31^ 2006	Described factors influencing mammography decisions	Age: 6 women were opposed to getting screened mammography; 3 mainly due to age. “I decided [not to go for mammography] because I am an old woman and nobody lives forever.” “I am old and it is not very important to me.”
Torke et al,^33^ 2013	Perceptions and experiences of screening decisions; described potential impact of factor on their decision-making process	Age: “If I’m 90 and I’m crippled up with arthritis, forget it. If I’m 85 and I’m crippled up with arthritis and I have memory problems and I can’t talk to people, forget it.”
		Living in a nursing home: “If I’m in a nursing home and the screening tests are coming up, no, I wouldn’t bother with it because my life is going to end…I’m going to die anyways.” “I don’t think none of that would influence me…They always want you to go to nursing homes.”
		Lack of insurance coverage: “That might entail a lot more than I can afford.” “It’s important to me, yes it is very important to me. But if my health was really at stake, I’d take a chance on maybe if they’d pay for it a little at a time, even, I would want to have it.”
Health and clinical		
Collins et al,^36^ 2010	Open questions about screening in women ≥70 y	Life expectancy: most influential factor for attending screening was to increase life expectancy.
		Quality of life: wish to maintain optimal quality of life; early detection also means major surgery and longer hospitalization can be avoided.
Dolezil et al,^37^ 2016	Open questions about utilization of early cancer screening	Targeted treatment: “That a specialist tells me accordingly, if everything is ok or that he also signals when he must explain anything in advance or but still, then if I was ready to respectively when it’s even also an unfavorable diagnosis, I’d do it anyway, so that he could tell me the truth and then I could get a doctor who could take the time and then discuss with me any further strategies.”
		Longer life: “That there’s time enough for the worst-case scenario to be recognized and then it can be handled accordingly. With that you can have another few years.”
Housten et al,^30^ 2018	Circumstances that would lead them to stop having mammograms	Other health problems: nearly two-thirds of participants reported they would continue even if diagnosed with a medical condition other than breast cancer: “Well I don’t think anything would make me stop. I can’t think of any ailments that would make me stop.”
		Memory problems: would not consider discontinuing even if they began experiencing severe memory problems. Instead, they would continue screening and believed their families would support this decision. “No [I would not stop mammograms] because [I would do] anything that is going to help me. Memory has nothing to do with…cancer.” “I have memory problems, they don’t stop me from having them [mammograms], you know.”
		Life expectancy <5 y: would continue even if they had <5 y to live. “Knowing that there might be a possibility, no matter what age you are or what year it is, that it could…[be cancer]…I would still, I believe at this point, want to have it [a mammogram] done.” “You fight for your life…Any way, anywhere….”
		Not be willing to undergo treatment for breast cancer: would continue screening even if they did not plan to undergo treatment. “No, I don’t think it [not willing to undergo treatment] would [stop me from getting mammograms].” However, others expected to undergo treatment if diagnosed; therefore, they had difficulty answering the question about how refusing treatment would influence their future screening behaviors. “Well, I would have treatment…I can’t [answer if I would stop screening].” “If I had it [cancer], I would not be a person who would not want it treated.”
Lewis et al,^38^ 2006	Attitudes toward continuing screening later in life	Deteriorating health, poor quality of life, or nearing death: “If I were going to die anyhow, from my heart etc, I would want to stop cancer screening.” “If I were doing poorly in every other way, I might say why bother.”
Pappadis et al,^39^ 2018	Explored influence of overdetection on cancer screening intentions	Comparison with other health conditions: Those who understood overdetection compared it with other conditions: “Some of them, it [prostate cancer] kills and some of them go on with their life and it doesn’t bother them a bit.”
Roy et al,^35^ 2020	Attitudes and perceptions of cancer screening and overscreening	Family history: “I always do my screening because I have a sister diagnosed with cancer, and because it could be in the genes, I always get checked. Just in case.” “I started doing my mammograms since I was 35 years old. I have a family history of breast cancer from my mom’s side. Two of my aunts died at a young age.”
Schoenborn et al,^13^ 2017	Explored considerations around the decision to stop screening	Health status: most believed health and functional status were important factors. “There are people much younger whose health is very poor, and people who are 80 and 90 whose health is very good, so age is not the only determining factor [in cancer screening] in my opinion.” When given example of healthy older person (would have been recommended to stop screening based on age), many supported continuing. Some viewed screening as way to evaluate poor health: “If a person is sick all the time any test they do has got to help, it can’t hurt…if they were really sick they’d probably need more tests.” When provided example of sick younger person (would have been recommended to screen based on age), many agreed stopping made sense: “Don’t do it. [Cancer] isn’t gonna be the thing that kills the people if they’ve got all those [health] problems.”
		Life expectancy: did not perceive life expectancy as being directly related to health status and age; perplexed when shown the Choosing Wisely statement “Don’t recommend cancer screening if patient is not likely to live 10 years.” All except 2 participants objected or questioned the statement. Reasons for objection included skepticism about life expectancy predictions, skepticism about screening’s lag time to benefit, and perceived negativity of the statement. One person described her doubt about the life expectancy prediction even if someone had multiple health problems: “How do you actually know the patient is not gonna live 10 years? I mean you look at it statistically I guess, you look and say…because she has this and this [health problem], she probably won’t live, but you never know. There’s always that one person who’s able to get over the hump.”
Schonberg et al,^31^ 2006	Described factors influencing mammography decisions	Health: “If I was dying from something else then I probably would not worry too much about breast cancer.”
		Functional status: “As long as I can get there.”
		Previous screening experience: “When I went for a mammogram, around me everybody was nervous and I decided that this is the time to stop.”
		Family history of breast disease: “I feel that because of my family history it is a good idea.”
		Personal history of breast disease: influenced women aged ≥80 y to screen; “I did have one biopsy…It was nothing…I just come back automatically once a year to be sure.”
		Validation of health: influenced women aged ≥80 y to screen
Torke et al,^33^ 2013	Questions about patient perceptions and experiences of screening decisions; described potential impact of factor on their decision-making process	Limited life expectancy: “Oh yeah. That would dictate whether the test would be important to take or not.” (factor to consider for stopping); “No, doctors don’t know how long you’re gonna live.” (not a consideration for stopping)
		Desire to live longer: “I want to stay on this earth as long as possible, and the best way to do it is to take these tests.” (continuing)
		Other health problems: “With all that’s wrong with you, I think I would get tired of going and getting something, because by that time…I’d have to have somebody to take me…I think I would just give up.” (consideration for stopping)
		Memory problems: “If your memory’s gone, and everything else, I’m thinking, there’s no point in having all those tests done.” (consideration for stopping)
		Family history: “I just feel like it’s something I need to do…because I have a family history of cancer.” (reason for continuing)
		Poor health or advanced age: “If I’m 90 and I’m crippled up with arthritis, forget it. If I’m 85 and I’m crippled up with arthritis and I have memory problems and I can’t talk to people, forget it.” (reason for stopping)
		Lack of family history: “I don’t have no history of cancer at all, of any kind, in my family. That’s what influences my decision.” (reason for stopping)
Psychological factors		
Collins et al,^36^ 2010	Open questions about screening in women ≥70 y	Peace of mind: “Just peace of mind really, just the hope that they’re not going to find anything, and if there was anything that they would, that it was early enough, for them to do something about it.”
		Agency in decision: Women believed in importance of right to choose for themselves. Although they would be willing to discuss the risks and benefits of breast screening with their general practitioner, they would not want them to make decisions on their behalf. Wanted increased information to allow them to make an informed decision.
Dolezil et al,^37^ 2016	Open questions about utilization of early cancer screening (quotations translated from German)	Habit: “I’ve been going to prostate screenings for 20 years.” “I do it regularly. I do this prostate screening yearly. The colonoscopy I do every three or four years. It’s always decided by the doctor…And I’ve now actually been doing that for over 20 years.”
		Fulfilling a duty: “When something happens, then it happens, that I can’t change. But I don’t want to blame myself or anyone else. You should have, you shouldn’t have.”
		Fear of screening participation: “Um it was fear. It was the pure fear of it, not, that it would get me too. And ah, I had three small kids at the time, so I also wanted to stay as long as possible with them, right?”
		Fear of screening nonparticipation: “Yes and I said that at the time, if you still have two years, ah, to live, the way she’s living, hair fallen out and everything else. Then that’s not what you want, then you’ll die and so, so I still think so today.”
		Disinterest: “Then I said after that that I had no interest in that anymore, I said, whenever you get sick, what happens.”
		No necessity: “What do they want from me? I feel really healthy, really good, food and drink tastes good and I really love women.”
		Early detection: “I hope, if something really happens, that I’ll be there at the right time and that something can still be done to prevent the *worst*. Yes, it’s good [spoken very softly]. That a good result comes out of it.”
		Reassurance: “I am then always more relaxed, must I say, when I go there and it still gives me something back, like, *I’m satisfied*. It *is all okay*, nothing more.”
		Conflicting information: “Let’s put it this way, for men you probably know PSA. It’s all controversial, health insurance companies write that, the doctors say that, so it’s a mess. From my point of view, *I am a technician…*if we had done something like that you’d be quite astonished.”
Gaehle et al,^29^ 2004	Perspectives on breast screening tools; barriers and facilitators to participation	Action stage: illustrated by statements such as “I don’t do much for myself as it is. I think, well, at least I can do that for myself.”
		Maintenance stage: a few women had the attitude, “Just do it” or “It’s the thing to do.”
		Discomfort: “It seems like they are trying to put your whole body in there.” “[It was] agony.” “I was sore for a week after.” “I had it yesterday and it still hurts today.” (barrier)
		Remembering to do it yearly (barrier)
Housten et al,^30^ 2018	Circumstances that would lead them to stop having mammograms	Routine: participants cited that routine was a reason to continue.
Lewis et al,^38^ 2006	Attitudes toward continuing screening later in life	Concerns about screening tests: “If screening methods were proven unreliable or if screening dangers outweigh the possible benefits.” “If I felt the test was unreliable or if early detection did not have much of an effect.”
Oliveira Leite et al,^34^ 2019	Older women’s perceptions about preventive cancer examination of cervix	Feelings and experiences of the elderly woman about the cervical cancer prevention examination: Despite the knowledge about the importance of the examination, in abstracting the feelings experienced by the elderly when they remembered to undergo this examination were highlights: the fear of the test result, nervousness, anxiety, and discomfort to perform it. “I get nervous, especially when I go to the nurse or the doctor.” “It’s like that, that feeling of fear, it’s not fear, but it’s like that anxiety.”
Pappadis et al,^39^ 2018	Explored influence of overdetection on cancer screening intentions	Varying desire to know about presence of cancer: Importance of knowing what was wrong and stating that it is better to know than not know was emphasized for those continuing. “If they find something, they just find something. It’s better to know than not know.” All who believed it was better to not to know also understood overdetection and chose to discontinue. Whereas others believed it was better to not know, because it was “not going to hurt her” and “she would not worry.”
		Right to decide: Several emphasized a personal decision should be made about having a mammogram and treatment: “It’s a choice that people just have to make. Right or wrong, you make your choice.”
		Necessity of screening older women: Support regular mammograms; several who did not understand overdetection believed older women should still get mammograms to be on the “safe side.” “Would have known if she went to get her screening.” A common subtheme among those who understood overdetection and decided not to undergo additional screening mammograms was “no symptoms, no mammograms.” “If you don’t have no symptoms, then there’s no reason to have the mammogram.”
		Resistance to concept of overdetection: negative persuasion, “It [overdetection] might encourage women not to get mammograms…and that could be a risk.” “How do you know? I think there’s no way of knowing it until you had a mammogram.” Only 5 of 59 women stated that the information about overdetection influenced their decision to receive a mammogram in the future, with all 5 stating they were less likely to screen. The remaining women stated that the information on overdetection would not influence their decisions about mammograms.
Roy et al,^35^ 2020	Attitudes and perceptions of cancer screening and overscreening	Importance of tailored and targeted education and information: “But after you get informed, you really think about it. The best thing out there is the information.” “Doctors will need to be clear with specifics about my health situation when explaining why they say not to have more cancer screenings.” Both English- and Spanish-speaking participants agreed that they would potentially be okay with screening cessation if they received specific, tailored information from doctor to justify why they do not need screening anymore. However, both groups were generally proscreening and mentioned wanting to seek a second opinion if their doctor recommended stopping screening.
		Negative perceptions and attitudes as barriers (pain, fear, stress, time, fatalism, cost): “I had a mammogram done and they squeezed my breast so bad that I almost lost my breath and was about to faint. Since that day, I don’t want to do any more mammograms.” “People are afraid of this test [i.e., colonoscopy].” “It’s better when you don’t know anything. You have a better life. Because if you got something, they are going to make you worry more, and your life will end more quickly. You will be miserable every day.” English-speaking groups brought up issue of stress and its potential negative impact on screening decision and health outcomes related to cancer diagnosis.
		Unawareness of potential risks of screening (eg, perforations, false positives): “I do not agree with a doctor telling me to not doing more cancer screening. I think we have the right to get the test done to prevent cancer. I think it will be always good to have the screenings done.”
		Importance of screening: “People are afraid of this test but it is important to do them.” “I think this person should still get the screening to see what is going on so he/she can feel better.”
Schonberg et al,^31^ 2006	Described factors influencing mammography decisions	Patient preferences: Taking care of oneself, “I think [mammography screening] is good for everybody. All women should find out about their bodies.’’ Rather not know: ‘‘Maybe the less I know the better.” (reason to not screen)
		Habit of screening: “I have become used to it, I have been having it for quite a few years now.”
		Perceived risk of breast cancer: “Once you reach 80, you do not have to worry.”
Swinney et al,^32^ 2011	Experiences, beliefs, and perspectives about breast cancer screening and risk-reduction behaviors	Avoiding finding out: Several participants shared they did not want to know if they “do have it [cancer].” They “want to be ignorant” rather than go to a health care practitioner and be diagnosed with cancer because they were afraid of the unknown consequences. “I can imagine my reaction [finding out] would just send me into a tipsy. I would just lose my mind…thinking about it. It would stress me out.”
		Fear or fear of disfigurement: Many participants expressed fears related to cancer. The most frequently expressed fear was of “cutting” the breast or total mastectomy. “Being diagnosed with breast cancer means automatically you’re going to have a breast taken off.”
		Beliefs about breast cancer: Family teachings and cultural beliefs about breast cancer informed many participants. One had 17 siblings and said that her mother taught every one of her daughters that hitting and squeezing one’s breasts can cause cancer. Others were taught by older relatives that breast cancer could result from not nursing a baby, as the milk would clog one’s breasts. Several believed that if you did get cancer, all surgical procedures should be avoided as cutting would expose the cancer to air and cause it to spread.
		Cancer as a death sentence: The participants had many ideas about cancer and how one gets the disease. However, the theme that consistently emerged from the transcripts was that once a woman “got the disease” or once “cancer got hold of you,” the outcome is death. “Most of the women that I know say it hurts too bad. Some people, they don’t want to know that they do have it. They just want to be ignorant from it than rather than go find out ’cause they’re afraid. Yeah, because cancer was always a death sentence.” On the other hand, some informants believed that a diagnosis of cancer was no longer always the death sentence it used to be.
Torke et al,^33^ 2013	Perceptions and experiences of screening decisions; described potential impact of factor on their decision-making process	Desire to prepare for end of life: “Because if I’ve got cancer…I want to know so that I can get ready with the Lord to go.” (continuing)
		Desire to gain knowledge: “I want to know. And if I didn’t keep getting them, I wouldn’t know.” (continuing)
		Desire to obtain treatment: “Try to get it taken care of, or let Dr S know about it.” (continuing)
		Early detection of cancer: “I would want them to do it as soon as I could for fear that if I did have it, they would be able to catch it before it spread too far.” (continuing)
		Habit: “Because I was getting [mammograms] every year. Well, I’ve been having them done periodically.” (continuing)
		Fear of cancer: “I feel like cancer’s about the worst thing you could have, and I think it would go over all the rest of [my other medical conditions].” (continuing)
		Reassurance: “It would give me peace of mind.” (continuing)
		Racial differences in risk: “I feel like all Black women should have it because different things happen to us.” (continuing)
		Screening no longer appropriate: “They didn’t do a Pap smear because there wasn’t nothing to check. That’s why I don’t have it anymore.” (stopping)
		Burdens of the test or burdens outweighing benefits: “I used to work at a hospital where we did colonoscopies and I don’t know what they do now for cleaning out the colon, but it wasn’t pleasant when I worked down there…drinking all that Colyte or whatever.” (consideration for stopping)
		Risks: “If they told me I was going to come in to some danger and I’m already not that well…I just say no, I don’t want to have it” (consideration for stopping). “They do tell you [about the risks]. And I think they should tell patients…it could happen…but usually don’t.” “I wouldn’t want to hear any risk.” (not consideration for stopping)
		Lack of benefit; life expectancy not long enough to benefit: “Why should I submit myself if I will not benefit from it?” (consideration for stopping)
		Statistical information: not relevant to individual decision-making; “I would not consider myself part of the statistics, and I think each individual person is different.”
Physicians		
Dolezil et al,^37^ 2016	Open questions about utilization of early cancer screening	Doctor recommendation: “It’s always decided by the doctor.” “Well our GP, who told back then that I should maybe go to a prostate screening when I’m at my age that I should do it. And then I went and did it.”
Gaehle et al,^29^ 2004	Perspectives on breast screening tools; facilitators and barriers to participation	Precontemplation phase: Physicians played a major role in whether these women participated in breast cancer screening or did not participate. “My doctor says I don’t need one.”
		Technician attitudes: “He fiddled with it a long time. I think he was just playing with my breast.” (barrier)
		Physician influence: Most women relied on their physician to order a yearly mammogram and then it was up to the woman to follow-through and have the test completed.
Housten et al,^30^ 2018	Circumstances that would lead them to stop having mammograms	Being told they would not live long enough to benefit: reluctant to consider changing screening behaviors based solely on doctors’ recommendation they would not live long enough to benefit. “No because they [doctors] don’t know. They don’t know when my expiration date…only that one [God] knows when my expiration date is up.” “No. It wouldn’t influence me, because nobody can’t say who going to live and who not going to live. That’s in God’s hands.”
		Being told screening would not extend life: would continue even if a physician told them that it would not increase their life expectancy; they might even question the doctors’ advice. “Just because that doctor says it does not mean that’s the gospel. If it’s not going to hurt you, you’re not taking any medicine, it’s just someone examining you. I would want to have it done.” “I just cannot imagine a doctor saying that a test would not prolong your life.”
Lewis et al,^38^ 2006	Attitudes toward continuing screening later in life	Doctor recommendation: “As far as I know I should continue, so unless my doctor says to stop I will continue, despite the pain.” “The doctors don’t think the colonoscopy is that necessary at my age, and I do what the doctor says.”
Oliveira Leite et al,^34^ 2019	Older women’s perceptions about preventive cancer examination of cervix	New patterns of sexuality in elderly women: an issue to be considered by women’s public health policies in the family health strategy. When questioned about the time of the last cervical cancer prevention examination, it was observed that most of them performed it from 3 to 10 y and one never did it, being the reason for this delay the lack of information given by health professionals about the importance its realization, as evidenced in the fragments: “It’s about 2 to 3 years old, which I don’t do, I really don’t because the doctor right here at the health center told me that I didn’t have to do more.” “The last time I did it was four years old or more.” “Nurse said you don’t have to do it anymore.” “The doctor said I didn’t need to do it.”
Pappadis et al,^39^ 2018	Explored the influence of overdetection on cancer screening intentions	Doctor recommendation “I think if the doctor told me that I needed to get a mammogram, I’d go get one…I don’t think you should have a closed mind at any age.”
		Second opinion: “I am going to ask my doctor and if I like it [opinion], I’ll do what she says and if I don’t I’ll do what I feel I need to do.”
Roy et al,^35^ 2020	Attitudes and perceptions of cancer screening and overscreening	Physician plays critical role: important for physician to communicate clearly, justify recommendations, foster trust, and tactfully provide information regarding health status, age, risks, family history, and screening history when recommending stopping screening. Varied opinions on whether they would accept a recommendation to stop screening. “I think even though the doctor suggest to stop cancer screening, I will still do them.” “I have a good relationship with my doctor. Whatever she recommends to do, I do it. She said I didn’t need the Pap smears because every time I had it, the results were good.” Overall, participants trusted doctor’s recommendation to screen, but were less trusting of doctor’s recommendation to stop screening. English-speaking participants more likely to be the ones who made final decision, Spanish-speaking participants said doctor would make decision for the most of the time.
Schoenborn et al,^13^ 2017	Explored considerations around the decision to stop screening	Physician recommendation: many said that they would view suggestion to stop screening from regular clinician as acceptable or positive. “I’d feel good that I didn’t need [another screening].” Some said they would think more highly of them: “If the doctor says to me we don’t have to do this no more I’d say: ‘Thank you very much Doc’…I probably [would] think more of him.” “I have all the confidence in her and if she told me to stop it I would stop.” Some were skeptical of suggestion to stop or said they would still insist on screening. Even among these, participants said that the clinician’s suggestion would not necessarily make them think less of the clinician or trust them less. “I told [my doctor] that I would want another [mammogram]. He said, ‘But at your age, 75, [we] don’t usually give another test.’ I said: ‘What difference does it make [what] my age is…I’m still human you know, why not another test?’ But he said, ‘You are fine, you ain’t got nothing to worry about.’ I said: ‘Well, I must be fine….’ He’s a good doctor and I trust him.”
Schonberg et al,^31^ 2006	Described factors influencing mammography decisions	Doctor recommendation: “No doctor has told me to have a mammogram lately, so I have not been bothered with that.”
Torke et al,^33^ 2013	Perceptions and experiences of screening decisions; described potential impact of factor on decision-making process	Active discouragement of screening by physician: Many would question recommendation to stop cancer screening or seek second opinion; disbelief that a physician would ever recommend stopping. “If my doctor said that he didn't think I'd benefit, I'd take his word.” “I think I might seek another doctor, get a second opinion.”
Social and system		
Collins et al,^36^ 2010	Open questions about screening in women ≥70 y	Invitation: “I’m very bad at remembering. If I had a reminder to say go on so and so date I’d be much better at keeping the appointment.”
Gaehle et al,^29^ 2004	Perspectives on breast screening tools; facilitators and barriers to participation	Experience of other women: knowledge of other women’s experiences was valuable for the women in this study. Several stories were told about women diagnosed with breast cancer who were acquaintances of the women in the focus groups.
		Media: Although it did not directly influence the women to specifically participate in any one form of breast cancer screening, all of the participant focus groups identified the many opportunities they have had to learn about breast cancer screening through media sources (eg, television programs, magazines, and news specials, which highlighted early, breast cancer detection methods). Women agreed with the statement, “This information is everywhere you look.” Several women mentioned local walks for breast cancer as well as specifically mentioning the Race for the Cure program.
		Family and friends: influenced participation for some of these women. “My daughter and her mother-in-law and I schedule our mammograms on the same day and same time. We go together and then go out for lunch.” This group of women seemed to attempt to make the experience more appealing by combining it with a multigenerational social event.
Housten et al,^30^ 2018	Circumstances that would lead them to stop having mammograms	Expert or governmental panel recommendations to discontinue: Skeptical of experts and governmental panels; reported that they would continue screening even if it conflicted with expert or panel recommendations. “Government panels aren’t always correct.” “If I wanted to have it, and I felt that I needed it, I would have it.” Many indicated they might consider discontinuing because of expert or panel recommendations. “It would probably depend upon the panel or whatever, because they wouldn’t know me personally. I’d still stick with my doctor.” “I suppose [I would consider recommendations by experts or governmental panels], but I am not sure I trust [them].”
		Family: participants cited that family obligation was reason to continue.
Roy et al,^35^ 2020	Attitudes and perceptions of cancer screening and overscreening	Family and friends: encouraged to get screened, provided different types of support (decision-making or instrumental support, such as driving to appointment). “I had a mammogram done about 2 years ago and my daughter recommended me to do it. She took me to the doctor.”
Schonberg et al,^31^ 2006	Described factors influencing mammography decisions	Family and friends: “The reason why I really started having mammograms [was] because my sister she died of cancer and I do not think she ever went for a mammogram.” “My daughter makes sure I get to all of my scheduled appointments.” (reason to continue only)
		Mailed reminder cards and access: “The hospital sends me a notice when it is time [for my mammogram] and that is about it.” (reason to continue only)
		The ease of actually getting a mammogram: “It is easy. I just come in and have it done and go home.”
Swinney et al,^32^ 2011	Experiences, beliefs, and perspectives about breast cancer screening and risk-reduction behaviors	Tending to one’s family: Many women in the focus groups viewed their role in their families as taking care of their family members and meeting life head on. They believed the well-being and care of their families took up most of their time and energy. Although the participants did not want to get breast cancer, they believed that taking care of their families came before taking care of themselves.
Torke et al,^33^ 2013	Perceptions and experiences of screening decisions; described potential impact of factor on their decision-making process	Burden on family and others: “I don’t like to be a burden or an interference with my son’s life because he has to learn how to live as an adult without his wife.” (stopping)
		Intergenerational equity: “There’s no real point to it, and I do think there is getting to be a burden on senior citizens who can’t really do very much to help, and they are costing more time and money to be spent on their problems, when small kids aren’t getting as much help as they need.” (stopping)
		Recommendation by independent experts or government panels: Negative responses: “I have heard so much of the government changing their minds on this…just like for example, coffee’s not good for you, then coffee’s right for you, you know…I don’t have too much faith in some of them.” (not considered for stopping)

### Demographic Factors

Nine studies^13,14,21,22,26,28,31,33,38^ found that older age was associated with the decision to forgo screening (eg, “If I got to be really old, I think I would say to heck with it. Like in my nineties.”). In contrast, another study^25^ found age was associated with older women’s decisions to both forgo and undergo breast screening. Women also held negative views toward upper age limits for breast screening eligibility in 1 UK study with moderate risk of bias.^36^

Quantitative studies consistently found that those with higher education^28^ or social class or income^21,28^ were more likely to undergo screening. Three quantitative studies^23,26,28^ and 1 qualitative study^33^ found that not having insurance (US) was associated with forgoing screening. However, 2 studies found no association of income or insurance with breast screening decisions.^22,24^ Two studies also found no association with race or ethnicity.^24,28^ Being married was associated with a greater likelihood of undergoing breast screening in 2 studies,^21,24^ but another study at moderate risk of bias found that marital status was not associated with cervical screening decisions.^28^ One qualitative study suggested living in a nursing home was not associated with the decision to forgo screening,^30^ but 3 studies^33,37,38^ (2 of which had risk of bias concerns^37,38^) highlighted that some individuals may be open to stopping for this reason.

### Health and Clinical Factors

The association of overall health status with screening decisions varied across studies. Qualitative studies^13,38^ suggested that older adults may consider their overall health status or health problems in their cancer screening decision-making. However, quantitative evidence^23,25,37,38^ suggested that this was not the majority view, and 3 other studies^28,30,36^ found that health status was not associated with cervical or breast screening decisions. More specific problems considered as reason to forgo screening included memory problems, “dying from something else,” or not being functionally able to undergo screening.^31,33,38^ Qualitative studies^13,31^ suggested screening was instead seen as a way to evaluate or validate health status or avoid major surgery or longer hospitalization as a result of early detection.^36^ Individual studies found that no physical disability (compared with severe disability),^21^ not having depression or anxiety,^21^ and greater quality of life increased the likelihood of choosing screening.^14^

The association of life expectancy with screening decisions was also inconsistent across studies. In a discrete choice experiment,^14^ participants were more likely to choose screening for a hypothetical patient with 5 or 10 years life expectancy than a patient with 1 year life expectancy (but 57.2% still chose screening for the latter), and a qualitative study^33^ found that some individuals may consider limited life expectancy as a reason to stop screening. However, 6 qualitative studies^13,30,33,35,37,38^ highlighted older adults’ resistance to using life expectancy to dictate or communicate about screening decisions. Instead, some thought that screening was a means of increasing life expectancy.^33,36,37^

Personal or family history of cancer consistently was associated with the decision to undergo screening in 5 studies.^25,28,31,33,35^ However, another study^22^ found that the majority of women thought they should continue breast screening even if they did not have any risk factors. Prior screening experience was associated with continued screening,^14,22,24,26^ but this was also reason to forgo breast screening.^25,31^ Screening to target treatment appropriately was expressed in 1 qualitative study (with moderate to high risk of bias),^37^ and women were also hesitant to stop breast screening even if they did not plan to undergo treatment.^30^ Women who had not had a hysterectomy were more likely to undergo cervical screening,^26^ and the number of visits to clinicians and exercise were not associated with breast cancer screening decisions.^24^

### Psychological Factors

Positive screening attitudes consistently were associated with the decision to undergo screening across many studies, including the importance of early detection and testing,^23,26,33,37^ belief in screening,^35,38,39^ just wanting to do it,^29^ fulfilling a duty,^37^ a way of taking care of oneself,^31^ and peace of mind.^27^ In 3 studies,^14,37,38^ many older adults agreed that they planned to get breast, prostate, and colorectal cancer screening for as long as they live. Similarly, negative screening attitudes consistently were associated with the decision to forgo screening across many studies, including simply not wanting screening,^26,36^ lack of benefit or appropriateness,^33,37^ disinterest,^37^ test discomfort or pain,^26,29,34,35,36^ embarrassment or privacy concerns,^36^ fatalism,^32,33,35^ the potential trouble obtaining insurance coverage if the individual is identified as high risk,^26^ and stress, time, or cost.^35^ Routine and habit also were associated with the decision to undergo screening,^23,25,30,37^ and forgetting was associated with forgoing screening.^29,36^

Perceived risk and fear of developing cancer were inconsistently operationalized and assessed across studies, meaning that findings were varied regarding their association with decision-making. High perceived risk was associated with continued screening,^22,24,26^ and low perceived risk was associated with forgoing screening,^25,31^ except in 1 study.^28^ Cancer fear, anxiety, and concern were associated with both the decision to undergo^22,33^ and forgo screening,^32^ except in 1 study.^26^ Fear of screening itself reduced the desire to screen,^32,33,34,37^ and fear of not being screened was associated with continued screening.^37^ Gaining knowledge or reassurance was associated with the decision to undergo screening,^23,25,27,33,36,37,39^ but not wanting to know also was associated with the decision to forgo screening.^31,32,34,39^

Risks of screening were also inconsistently assessed, and results varied across the included studies regarding their influence. Qualitative findings suggested that screening risks may be associated with the decision to forgo screening^33,38^ as well as knowledge of overdetection in 1 quantitative study.^39^ However, 3 studies found low awareness of potential risks of screening,^35^ low worry about risks of breast screening (low to moderate risk of bias),^36^ and resistance to the concept of overdetection.^39^

The association of screening knowledge or information with decision-making was extremely varied across included studies. Awareness of US Medicare coverage^22^ and the starting age for screening^24^ was associated with continued breast screening, and not knowing about access beyond age 70 years was a reason for not screening (low to moderate risk of bias).^36^ Receiving information was generally expressed by older adults as influential,^23^ and many specifically desired information about the possibility of false-positive findings.^23,37^ Older adults desired information for agency in the decision,^35,36,39^ but looking for cancer information was not associated with breast screening decisions,^24^ and statistical information was not deemed relevant.^33^ However, some older adults expressed a desire for specific, tailored information to justify a recommendation to stop screening.^35^

### Physician Factors

Seven studies^23,24,25,26,29,37,39^ consistently found that a physician’s recommendation to screen was associated with the decision to undergo screening. Seven other studies^13,14,25,29,34,35,38^ also found that a physician’s recommendation against screening was associated with the decision to forgo screening. In 1 study,^27^ discussing the disadvantages of screening was associated with stopping colorectal screening. Not receiving a recommendation was also reason to forgo breast screening for some individuals.^31^ However, qualitative studies highlighted more nuance surrounding the influence of a physician’s recommendation against screening. Some older adults reported they may respond negatively to a recommendation against screening; they were skeptical, especially when justified with life expectancy,^13,30^ desired a second opinion,^33,35^ or continued screening anyway; 1 study^38^ found that 43% of participants still desired screening despite receiving a recommendation to stop.

Believing that the physician makes important decisions and trusting them to make the best decision on their patients’ behalf were not associated with cervical screening decisions.^24^ One study^38^ also found that one-half of the participants thought that physicians know for certain whether cancer screening helps people older than 70 years (low to moderate risk of bias).

### Social and Societal and System Factors

Of the studies that examined the influence of family, most (6 studies) found that family was associated with the decision to undergo screening (including wives, daughters, mothers, and mothers-in-law, specifically for breast screening),^23,25,29,30,31,35^ but 3 studies^32,33,36^ found that family was associated with the decision to forgo screening because of older adults feeling they would become a burden^33,36^ or that they needed to care for family instead of themselves.^32^ One study^26^ specifically found that the perceived positive impact of screening for the family was not associated with the intent to undergo cervical screening. Five studies consistently reported the association of friends with older adults’ decisions to undergo breast and prostate screening.^23,25,29,31,35^

Invitations and reminders consistently were associated with the decision to undergo breast screening (in the United Kingdom and US),^25,31,36^ but skepticism toward government recommendations to discontinue screening was also expressed in 2 studies.^30,33^ However, it was unclear how government recommendations were associated with decision-making. The association of media with undergoing breast screening was also expressed in 2 studies.^25,29^ Single studies found that access to transportation was associated with prostate cancer screening decisions,^23^ transport difficulties specifically discouraged breast cancer screening attendance,^36^ and ease of access was associated with women’s decisions to undergo breast cancer screening.^31^ Finally, societal costs and resource waste were reasons not to screen in 2 studies,^33,38^ but this may be the minority view because only 30% of participants agreed with the statement that “Screening for cancer in people over the age of 70 may waste healthcare time and money.”^38^

## Discussion

This systematic review synthesizes study findings regarding the factors associated with older adults’ cancer screening decisions. It builds on previous work in younger populations by including study designs that summarize how commonly studied factors (eg, personal or family history of cancer), psychosocial factors, and factors of more relevance to older people (eg, life expectancy and health status) objectively and subjectively are associated with older adults’ cancer screening decisions. The findings reveal a substantial gap in studies conducted outside the US, which is important given that many health systems vary considerably from the US health system.

Our findings highlight the degree to which aging-related factors associated with the likelihood of benefit and harms of cancer screening are associated with older adults’ decision-making. First, despite older age consistently being associated with older adults forgoing screening, recent US evidence highlights high rates of screening beyond recommended ages.^10^ Qualitative studies suggest that some older adults find the idea of no longer needing screening when much older more intuitive than stopping at a younger or older age (eg, “If I’m 90” or “dying from something else” vs 75 years). This may be because of widespread information that age is the greatest nonreversible factor associated with the risk of cancer. Second, although limited life expectancy is also associated with lower screening participation,^40,41,42,43,44^ rates of screening in older adults with limited life expectancy are still high.^9,44,45,46^ Findings from this review suggest that life expectancy may not be relevant to some older adults’ screening decisions. A recent survey of veterans (aged ≥50 years) supports this finding, as stopping screening for colorectal cancer because of age and life expectancy did not resonate.^47^ Finally, some older people may not consider other health problems as relevant to their screening decisions and, instead, could view screening as a means of validating their health. Relevant studies not included in this review similarly suggest that older people may see discontinuing screening as giving up^48^ and that being invited to participate in cancer screening symbolically demonstrates continued investment in their health.^49^ Although aging-related concepts are challenging to communicate, older people must be counseled about the reduced benefit and increased chance of harm from screening associated with limited life expectancy and worsening health to make better quality screening decisions.

Many psychosocial factors were consistently associated with older adults’ screening decisions across studies. Many of these factors should not be the main reason for screening decisions (eg, routine or habit and reassurance) and are likely substantial hindrances for older adults in considering the factors that should be considered to make better quality decisions (ie, life expectancy and overall health). One study^14^ specifically found attitudes toward screening are better at estimating older adults’ screening decisions than age and life expectancy, highlighting that an individual’s intrinsic attitude to screening needs to be understood when trying to foster informed decision-making. Given that aging-related factors are commonly incorporated into screening guidelines, future research should examine how physicians can sensitively communicate with patients regarding these factors in light of their screening attitudes. Our review suggests that some patients may appreciate a recommendation to stop cancer screening, especially if it is individually tailored and justified, but others may not. Although older adults may prefer a recommendation statement that incorporates health status,^50^ our Australian experimental study^51^ found that a more-confronting statement incorporating life expectancy could be more effective than a more-preferred statement incorporating health status in reducing screening intention without having adverse outcomes. More research with larger community samples is needed to examine how older adults respond to recommendations against screening and information about potential harms. Responses may differ according to different patient characteristics (eg, screening attitudes, knowledge, patient-physician relationship, and perceived risk) and the way it is communicated. Some older adults may also respond negatively to government recommendations against screening, perhaps because of the belief that it would be a cost-saving measure.^52^ This warrants further exploration across countries where differences in reimbursement for screening may differentially affect decision-making.

### Limitations

Our review was limited as we chose not to perform a meta-analysis because of the wide variability in designs and outcomes and explanatory variables in the included studies. Instead, we used narrative synthesis to present findings given that estimating the relative magnitude of effect sizes for these factors was not the aim. However, this review builds on existing narrative reviews by using a systematic approach and including a range of study designs with results presented narratively, meaning the nuance of the data could still be captured, as well as the consistencies and inconsistences across studies. Two independent reviewers conducted screening, data extraction, and risk of bias assessment, and gray literature (eg, theses) and non-English studies were searched and included. Another limitation was that the larger scope meant that studies examining combinations of breast, prostate, colorectal, or cervical cancer screening decisions were included, making it difficult to identify trends or differences across separate screening decisions. However, it was important to be more inclusive given the similarly high rates of screening evident across all 4 screening types, especially in the US,^9^ and the limited recent research in this field. Only 1 study exclusively included men, 7 studies that assessed multiple screening types included mostly women, and only 1 study analyzed sex-specific differences in decision-making (and found no differences). Therefore, future studies should examine sex- and screening-specific differences in decision-making. Large, nationally representative, cross-sectional surveys in different countries would also be useful to guide the targeting of communication strategies to support informed decision-making. More specifically, associations between demographic and health characteristics, screening attitudes and knowledge, and the extent to which factors such as health status, life expectancy, and personal risk are considered in older adults’ decision-making should be examined. An international comparison should also be conducted to explore differences between countries with and without nationally funded cancer screening programs (eg, US vs Australia).

## Conclusions

Older adults should consider aging-related factors such as life expectancy and overall health status in their cancer screening decisions, as these factors have important implications for their own personal chance of benefiting from screening and chance of adverse outcomes on health and quality of life due to short-term physical and psychological harm. Currently, their existing screening beliefs may run counter to these concepts, likely hindering efforts to support informed decision-making, and decision support or education about benefits and harms may not be enough to change screening intentions or behavior.^48,53^ Communication strategies ought to address these underlying screening perceptions and beliefs given older adults’ longer term exposure to positive screening messaging and the adverse impact cancer screening can have on their quality of remaining life.

## Notes

Supplement. **eTable 1.** Search Strategy **eTable 2.** Study Quality Appraisal Using Joanna Briggs Institute Critical Appraisal Checklists (Quantitative Studies) **eTable 3.** Study Quality Appraisal Using Joanna Briggs Institute Critical Appraisal Checklists (Qualitative Studies) **eAppendix.** Results of Risk of Bias Assessment
eReferences
