# Supplementary material for: Patient-Reported Factors Associated With Older Adults’ Cancer Screening Decision-making: A Systematic Review
Source: JAMA Netw Open. 2021 Nov 8;4(11):e2133406. doi: 10.1001/jamanetworkopen.2021.33406 (PMC8576581; doi:10.1001/jamanetworkopen.2021.33406)
Supplement: Supplement. — eTable 1. Search Strategy eTable 2. Study Quality Appraisal Using Joanna Briggs Institute Critical Appraisal Checklists (Quantitative Studies) eTable 3. Study Quality Appraisal Using Joanna Briggs Institute Critical Appraisal Checklists (Qualitative Studies) eAppendix. Results of Risk of Bias Assessment eReferences [file jamanetwopen-e2133406-s001.pdf]

Supplemental Online Content

Smith J, Dodd RH, Gainey KM, et al. Patient-reported factors associated with older adults' cancer screening decision-making: a systematic review. *JAMA Netw Open*. 2021;4(11):e2133406. doi:10.1001/jamanetworkopen.2021.33406

eTable 1. Search Strategy

eTable 2. Study Quality Appraisal Using Joanna Briggs Institute Critical Appraisal Checklists (Quantitative Studies)

eTable 3. Study Quality Appraisal Using Joanna Briggs Institute Critical Appraisal Checklists (Qualitative Studies)

eAppendix. Results of Risk of Bias Assessment

eReferences

This supplemental material has been provided by the authors to give readers additional information about their work.

© 2021 Smith J et al. *JAMA Network Open.*

eTable 1. Search Strategy

OVID Medline & Pre-Medline	Embase	PsycINFO	CINAHL
1. geriatrics/<br>2. geriatric*.tw.<br>3. older*.tw.<br>4. elderly.tw.<br>5. over 65.tw.<br>6. senior*.tw.<br>7. pensioner*.tw.<br>8. veteran*.tw.<br>9. 1 or 2 or 3 or 4 or 5 or 6 or 7 or 8<br>10. cancer/<br>11. cancer.tw.<br>12. neoplasms/<br>13. tumo?r*.tw.<br>14. breast neoplasms/<br>15. colonic neoplasms/<br>16. prostatic neoplasms/<br>17. uterine cervical neoplasms/<br>18. 10 or 11 or 12 or 13 or 14 or 15 or 16 or 17<br>19. mass screening/<br>20. early detection of cancer/<br>21. (screening* adj3 cancer).tw.<br>22. mammogra*.tw.<br>23. PSA test*.tw.<br>24. prostate-specific antigen.tw.<br>25. colonoscop*.tw.<br>26. FOBT.tw.<br>27. f?ecal occult blood test.tw.<br>28. pap smear*.tw.<br>29. pap test*.tw.<br>30. human papillomavirus.tw.<br>31. HPV test*.tw.<br>32. cervical screening test*.tw.<br>33. 19 or 20 or 21 or 22 or 23 or 24 or 25 or 26 or 27 or 28 or 29 or 30 or 31 or 32<br>34. decision making/<br>35. decision*.tw.<br>36. attitude/<br>37. attitude*.tw.<br>38. intention/<br>39. intention*.tw.<br>40. patient preference/<br>41. view*.tw.<br>42. perspective*.tw.<br>43. opinion*.tw.<br>44. 34 or 35 or 36 or 37 or 38 or 39 or 40 or 41 or 42 or 43<br>45. 9 and 18 and 33 and 44<br>46. limit 45 to yr=”2000-2021”	1. geriatrics/<br>2. geriatric*.tw.<br>3. older*.tw.<br>4. elderly.tw.<br>5. over 65.tw.<br>6. senior*.tw.<br>7. pensioner*.tw.<br>8. veteran*.tw.<br>9. 1 or 2 or 3 or 4 or 5 or 6 or 7 or 8<br>10. cancer/<br>11. cancer.tw.<br>12. neoplasm/<br>13. tumo?r*.tw.<br>14. breast cancer/<br>15. colorectal cancer/<br>16. prostate cancer/<br>17. cervical cancer/<br>18. 10 or 11 or 12 or 13 or 14 or 15 or 16 or 17<br>19. cancer screening/<br>20. mass screening/<br>21. early cancer diagnosis/<br>22. (screening* adj3 cancer).tw.<br>23. mammogra*.tw.<br>24. PSA test*.tw.<br>25. prostate-specific antigen.tw.<br>26. colonoscop*.tw.<br>27. FOBT.tw.<br>28. f?ecal occult blood test*.tw.<br>29. cervical screening test*.tw.<br>30. pap smear*.tw.<br>31. pap test*.tw.<br>32. human papillomavirus.tw.<br>33. HPV test*.tw.<br>34. 19 or 20 or 21 or 22 or 23 or 24 or 25 or 26 or 27 or 28 or 29 or 30 or 31 or 32 or 33<br>35. decision making/<br>36. decision*.tw.<br>37. patient attitude/<br>38. attitude*.tw.<br>39. intention*.tw.<br>40. patient preference/<br>41. view*.tw.<br>42. perspective*.tw.<br>43. opinion*.tw.<br>44. 36 or 37 or 38 or 39 or 40 or 41 or 42 or 43 or 44<br>45. 9 and 18 and 34 and 44<br>46. limit 45 to yr=”2000-2021”	1. geriatrics/<br>2. geriatric*.tw.<br>3. older*.tw.<br>4. elderly.tw.<br>5. over 65.tw.<br>6. senior*.tw.<br>7. pensioner*.tw.<br>8. veteran*.tw.<br>9. 1 or 2 or 3 or 4 or 5 or 6 or 7 or 8<br>10. cancer.tw.<br>11. neoplasms/<br>12. tumo?r*.tw.<br>13. 10 or 11 or 12<br>14. cancer screening/<br>15. (screening* adj3 cancer).tw.<br>16. mammogra*.tw.<br>17. PSA test*.tw.<br>18. prostate-specific antigen.tw.<br>19. colonoscop*.tw.<br>20. FOBT.tw.<br>21. f?ecal occult blood test*.tw.<br>22. cervical screening test*.tw.<br>23. pap smear*.tw.<br>24. pap test*.tw.<br>25. human papillomavirus.tw.<br>26. HPV test*.tw.<br>27. 14 or 15 or 16 or 17 or 18 or 19 or 20 or 21 or 22 or 23 or 24 or 25 or 26<br>28. decision making/<br>29. decision*.tw.<br>30. attitude*.tw.<br>31. intention/<br>32. intention*.tw.<br>33. view*.tw.<br>34. perspective*.tw.<br>35. opinion*.tw.<br>36. 28 or 29 or 30 or 31 or 32 or 33 or 34 or 35<br>37. 9 and 13 and 27 and 36<br>38. limit 37 to yr=”2000-2021”	S1 (MH “Geriatrics”)<br>S2 geriatric*<br>S3 older*<br>S4 elderly<br>S5 over 65<br>S6 senior*<br>S7 pensioner*<br>S8 veteran*<br>S9 S1 or S2 or S3 or S4 or S5 or S6 or S7 or S8<br>S10 (MH “Neoplasms”)<br>S11 cancer<br>S12 tumo?r*<br>S13 (MH “Breast Neoplasms”)<br>S14 (MH “Colorectal Neoplasms”)<br>S15 (MH “Prostatic Neoplasms”)<br>S16 (MH “Cervix Neoplasms”)<br>S17 S10 or S11 or S12 or S13 or S14 or S15 or S16<br>S18 (MH “Cancer Screening”)<br>S19 screening* N3 cancer<br>S20 mammogra*<br>S21 PSA test*<br>S22 prostate-specific antigen<br>S23 colonoscop*<br>S24 FOBT<br>S25 f?ecal occult blood test*<br>S26 cervical screening test*<br>S27 pap smear*<br>S28 pap test*<br>S29 human papillomavirus<br>S30 HPV test*<br>S31 S18 or S19 or S20 or S21 or S22 or S23 or S24 or S25 or S26 or S27 or S28 or S29 or S30<br>S32 (MH “Decision Making”)<br>S33 decision*<br>S34 (MH “Attitude”)<br>S35 attitude*<br>S36 (MH “Intention”)<br>S37 intention*<br>S38 (MH “Patient Preference”)<br>S39 view*<br>S40 perspective*<br>S41 opinion*<br>S42 S32 or S33 or S34 or S35 or S36 or S37 or S38 or S39 or S40 or S41<br>S43 S9 and S17 and S31 and S42

© 2021 Smith J et al. *JAMA Network Open.*

eTable 2. Study Quality Appraisal Using Joanna Briggs Institute Critical Appraisal Checklists (Quantitative Studies)

**Checklist for Cross-sectional Studies (9 items)**										
**Study (author, year)**	**CHECKLIST ITEMS**									**OVERALL APPRAISAL**
	**Was the sample frame appropriate to address the target population?**	**Were study participants sampled in an appropriate way?**	**Was the sample size adequate?**	**Were the study subjects and setting described in detail?**	**Was the data analysis conducted with sufficient coverage of the identified sample?**	**Were valid methods used for the identification of the condition?**	**Was the condition measured in a standard, reliable way for all participants?**	**Was there appropriate statistical analysis?**	**Was the response rate adequate, and if not, was the low response rate managed appropriately?**	
Collins et al., 2010*	?	+	+	+	?	+	+	+	-	Moderate
Dolezil et al., 2016*	?	+	?	+	+	+	?	?	-	High
Edwards et al., 2000	+	+	?	+	+	?	+	+	+	Low
Eisner et al., 2002	+	+	?	+	+	+	+	+	?	Low
Fairfield et al., 2015	+	+	+	+	?	?	+	+	-	Moderate
Gregory et al., 2007	?	+	?	+	?	+	+	+	+	Low
Lewis et al., 2006*	?	+	?	+	?	+	+	+	+	Low
Madadi et al., 2014	+	+	?	+	+	+	+	+	+	Low
Sawaya et al., 2009	+	+	?	+	?	+	?	+	-	Moderate
Schoenborn et al., 2019	+	+	?	+	+	+	+	+	+	Low
Schonberg et al., 2007	?	+	?	+	+	+	+	+	+	Low
Zhang et al., 2007	?	+	?	+	+	+	+	+	+	Low

*Mixed methods study
(+) met criteria, (-) did not meet criteria, (?) unclear
Low: ≤2 domains of concern or ≤3 domains unclear, moderate: 3-4 domains of concern where at least one domain is not fulfilled (-) or high: ≥5 domains of concern

© 2021 Smith J et al. *JAMA Network Open.*

eTable 3. Study Quality Appraisal Using Joanna Briggs Institute Critical Appraisal Checklists (Qualitative Studies)

Checklist for Qualitative Studies (10 items)											
**Study (author, year)**	**CHECKLIST ITEMS**										**OVERALL APPRAISAL**
	**Is there congruity between the stated philosophical perspective and the research methodology?**	**Is there congruity between the research methodology and the research question or objectives?**	**Is there congruity between the research methodology and the methods used to collect data?**	**Is there congruity between the research methodology and the representation and analysis of data?**	**Is there congruity between the research methodology and the interpretation of results?**	**Is there a statement locating the researcher culturally or theoretically?**	**Is the influence of the researcher on the research, and vice-versa, addressed?**	**Are participants, and their voices, adequately represented?**	**Is the research ethical according to current criteria or, for recent studies, and is there evidence of ethical approval by an appropriate body?**	**Do the conclusions drawn in the research report flow from the analysis, or interpretation, of the data?**	
Collins et al., 2010*	+	+	+	+	+	-	-	+	+	+	Low
Dolezil et al., 2016*	?	+	+	+	+	-	-	+	+	+	Moderate
Gaehle et al., 2004	+	+	+	+	+	-	-	+	+	+	Low
Housten et al., 2018	+	+	+	+	+	-	-	+	+	+	Low
Lewis et al., 2006*	?	+	+	+	+	-	?	+	+	+	Moderate
Oliveira Leite et al., 2019	+	+	+	-	+	-	-	+	+	+	Moderate
Pappadis et al., 2018*	+	+	+	+	+	-	+	+	+	+	Low
Roy et al., 2020	-	+	+	+	+	-	-	+	+	+	Moderate
Schoenborn et al., 2017	-	+	+	+	+	-	-	+	+	+	Moderate
Schonberg et al., 2006	-	+	+	+	+	-	?	+	+	+	Moderate
Swinney et al., 2011	+	+	+	+	+	-	-	+	+	+	Low
Torke et al., 2013	+	+	+	+	+	-	+	+	+	+	Low

*Mixed methods study
(+) met criteria, (-) did not meet criteria, (?) unclear
Low: ≤2 domains of concern or ≤3 domains unclear, moderate: 3-4 domains of concern where at least one domain is not fulfilled (-) or high: ≥5 domains of concern

© 2021 Smith J et al. *JAMA Network Open.*

eAppendix. Results of Risk of Bias Assessment

Seven of nine cross-sectional studies had low risk of bias[1-7] and two had moderate risk.[8,9] Four of eight qualitative studies had low risk of bias[10-13] and four had moderate risk.[14-17] Three of four mixed-methods studies were assessed using both the cross-sectional and qualitative checklist; two had low-moderate risk of bias[18,19] and one had moderate-high risk.[20] The remaining mixed-methods study had low risk of bias after being assessed using the qualitative checklist as it did not incorporate a quantitative survey.[21] Main concerns in cross-sectional quantitative studies were due to sampling (inadequate sample size or no sample size calculation in 10/12 studies, lack of representative samples in 6/12 studies) and inappropriate management of low response rates (5/12 studies). For qualitative studies, concerns were mostly due to lack of acknowledgement of the location of the researcher culturally or theoretically (12/12 studies), as well as their influence on the research (10/12 studies).

© 2021 Smith J et al. *JAMA Network Open.*

eReferences

1. Edwards NIJ, D. A. Uptake of breast cancer screening in older women. *Age and Ageing.* 2000;29(2):131-135.

2. Madadi MZ, Shengfan: Yeary, Karen H. Kim: Henderson, Louise M. Analyzing factors associated with women's attitudes and behaviors toward screening mammography using design-based logistic regression. *Breast cancer research and treatment.* 2014;144(1):193-204.

3. Eisner EJZ, Eric G.: Goodman, Nina: Macario, Everly. Knowledge, attitudes, and behavior of women ages 65 and older on mammography screening and Medicare: results of a national survey. *Women & health.* 2002;36(4):1-18.

4. Gregory DJ. Iowa men's decision-making process for prostate cancer prevention via screening with the Prostate-Specific Antigen (PSA) test. *Dissertation Abstracts International: Section B: The Sciences and Engineering.* 2007;68(3-B):1590.

5. Schoenborn NLX, Q. L.: Pollack, C. E.: Janssen, E. M.: Bridges, J. F. P.: Wolff, A. C.: Boyd, C. M. Demographic, health, and attitudinal factors predictive of cancer screening decisions in older adults. *Preventive Medicine Reports.* 2019;13:244-248.

6. Schonberg MAM, Ellen P.: York, Meghan: Davis, Roger B.: Marcantonio, Edward R. Factors influencing elderly women's mammography screening decisions: implications for counseling. *BMC geriatrics.* 2007;7:26.

7. Zhang YB, T. F.: Rohrer, J. E. Correlates of Intent to Seek Unnecessary Pap Tests Among Elderly Women. *Women's Health Issues.* 2007;17(6):351-359.

8. Fairfield KMG, Bethany S.: Levin, Carrie A.: Stringfellow, Vickie: Wierman, Heidi R.: McNaughton-Collins, Mary. Decisions about medication use and cancer screening across age groups in the United States. *Patient education and counseling.* 2015;98(3):338-343.

© 2021 Smith J et al. *JAMA Network Open.*

9. Sawaya GFI-S, A. Yuri: Kim, Sue: Wong, Sabrina T.: Huang, Alison J.: Washington, A. Eugene: Perez-Stable, Eliseo J. Ending cervical cancer screening: attitudes and beliefs from ethnically diverse older women. *American journal of obstetrics and gynecology.* 2009;200(1):40.e41-47.

10. Gaehle KE. *A conceptual understanding of breast cancer screening practices of older women between 65--84 years of age*, Saint Louis University; 2004.

11. Housten AJP, Monique R.: Krishnan, Shilpa: Weller, Susan C.: Giordano, Sharon H.: Bevers, Therese B.: Volk, Robert J.: Hoover, Diana S. Resistance to discontinuing breast cancer screening in older women: A qualitative study. *Psycho-oncology.* 2018;27(6):1635-1641.

12. Swinney JED, M. T. Older African American women's beliefs, attitudes, and behaviors about breast cancer. *Research in gerontological nursing.* 2011;4(1):9-18.

13. Torke AMS, Peter H.: Holtz, Laura R.: Montz, Kianna: Sachs, Greg A. Older adults and forgoing cancer screening: "I think it would be strange". *JAMA internal medicine.* 2013;173(7):526-531.

14. Oliveira Leite BON, Cleide Roseli: Vieira de Oliveira, Valdira: Alves Barbosa, Romana Aparecida: Santos Souza, Meriele: Barbosa Teles, Mariza Alves. The Elderly Women's Perception of Cervical Cancer Prevention Examination. *Revista de Pesquisa: Cuidado e Fundamental.* 2019;11(5):1347-1352.

15. Schoenborn NLL, Kimberley: Pollack, Craig E.: Armacost, Karen: Dy, Sydney M.: Bridges, John F. P.: Qian-Li, Xue: Wolff, Antonio C.: Boyd, Cynthia: Xue, Qian-Li. Older Adults' Views and Communication Preferences About Cancer Screening Cessation. *JAMA Internal Medicine.* 2017;177(8):1121-1128.

16. Schonberg MAR, R. A.: McCarthy, E. P.: Marcantonio, E. R. Decision making and counseling around mammography screening for women aged 80 or older. *Journal of General Internal Medicine.* 2006;21(9):979-985.

© 2021 Smith J et al. *JAMA Network Open.*

17. Roy S, Moss JL, Rodriguez-Colon SM, et al. Examining Older Adults' Attitudes and Perceptions of Cancer Screening and Overscreening: A Qualitative Study. *Journal of Primary Care and Community Health.* 2020;11.

18. Collins KW, M.: Reed, M. W.: Walters, S. J.: Robinson, T.: Madan, J.: Green, T.: Cocker, H.: Wyld, L. The views of older women towards mammographic screening: a qualitative and quantitative study. *British journal of cancer.* 2010;102(10):1461-1467.

19. Lewis CLK, Christine E.: Amick, Halle R.: Watson, Lea C.: Bynum, Debra L.: Walter, Louise C.: Pignone, Michael P. Older adults' attitudes about continuing cancer screening later in life: a pilot study interviewing residents of two continuing care communities. *BMC geriatrics.* 2006;6:10.

20. Dolezil DH, Annekathrin: Jahnke, Kristine: Thonack, Jens: Loffler, Christin: Schmidt, Carsten Oliver: Chenot, Jean-Francois. [Cancer screening in the elderly: Explorative mixed methods study]. *Krebsfruherkennungsuntersuchungen im hohen Alter: Explorative "Mixed-methods"-Studie.* 2016;49(1):44-51.

21. Pappadis MRV, Robert J.: Krishnan, Shilpa: Weller, Susan C.: Jaramillo, Elizabeth: Hoover, Diana Stewart: Giordano, Sharon H.: Tan, Alai: Sheffield, Kristin M.: Housten, Ashley J.: Goodwin, James S. Perceptions of overdetection of breast cancer among women 70 years of age and older in the USA: a mixed-methods analysis. *BMJ open.* 2018;8(6):e022138.

© 2021 Smith J et al. *JAMA Network Open.*
